# Widespread Impact of Chromosomal Inversions on Gene Expression Uncovers Robustness via Phenotypic Buffering

**DOI:** 10.1093/molbev/msw045

**Published:** 2016-02-28

**Authors:** Samina Naseeb, Zorana Carter, David Minnis, Ian Donaldson, Leo Zeef, Daniela Delneri

**Affiliations:** ^1^Computational and Evolutionary Biology Research Theme, Faculty of Life Sciences, University of Manchester, Manchester, United Kingdom

**Keywords:** inversions, fitness, genome organisation, *Saccharomyces cerevisiae*.

## Abstract

The nonrandom gene organization in eukaryotes plays a significant role in genome evolution and function. Chromosomal structural changes impact meiotic fitness and, in several organisms, are associated with speciation and rapid adaptation to different environments. Small sized chromosomal inversions, encompassing few genes, are pervasive in *Saccharomyces* “sensu stricto” species, while larger inversions are less common in yeasts compared with higher eukaryotes. To explore the effect of gene order on phenotype, reproductive isolation, and gene expression, we engineered 16 *Saccharomyces cerevisiae* strains carrying all possible paracentric and pericentric inversions between Ty1 elements, a natural substrate for rearrangements. We found that 4 inversions were lethal, while the other 12 did not show any fitness advantage or disadvantage in rich and minimal media. At meiosis, only a weak negative correlation with fitness was seen with the size of the inverted region. However, significantly lower fertility was seen in heterozygote invertant strains carrying recombination hotspots within the breakpoints. Altered transcription was observed throughout the genome rather than being overrepresented within the inversions. In spite of the large difference in gene expression in the inverted strains, mitotic fitness was not impaired in the majority of the 94 conditions tested, indicating that the robustness of the expression network buffers the deleterious effects of structural changes in several environments. Overall, our results support the notion that transcriptional changes may compensate for Ty-mediated rearrangements resulting in the maintenance of a constant phenotype, and suggest that large inversions in yeast are unlikely to be a selectable trait during vegetative growth.

## Introduction

Studies carried out in several species (i.e., humans, Drosophila, and yeast) have shown that eukaryotes have a highly ordered genome organization, which is crucial for gene regulation (Cho et al. 1998; Csink et al. 2002; Gierman et al. 2007). The genome is organized in the form of coexpressed gene clusters (Boutanaev et al. 2002; Singer et al. 2005; Lercher and Hurst 2006; Elliott et al. 2013), functionally related genes (Hurst et al. 2004), clusters of genes encoding protein complexes (Teichmann and Veitia 2004), and gene pairs with bidirectional promoters (Cohen et al. 2000; Xu et al. 2009). It has been shown that *Saccharomyces* *cerevisiae*/*Candida* *glabrata* group possess relatively lower genomic stability compared with the *K. lactis*/*A. gossypii* lineage (Fischer et al. 2006). The chromosomal organization between the genomes of *Saccharomyces* “*sensu stricto*” species has been disrupted not only due to a massive gene loss that occurred following the whole-genome duplication event (Wolfe and Shields 1997) but also due to the large rearrangements (Fischer et al. 2000, 2001; Kellis et al. 2003). Chromosomal alterations such as translocations, deletions, duplications, and inversions are an integral part of genome evolution and scientists have been trying to understand their effect on transcription, fitness, and speciation processes (Seoighe et al. 2000; Fischer et al. 2001; Britten 2002; Colson et al. 2004). Studies on chromosomal translocations in *S.* *cerevisiae* (Colson et al. 2004) and on translocations and inversions in *Schizosaccharomyces pombe* (Brown et al. 2011; Avelar et al. 2013) showed that these rearrangements can confer fitness alterations in specific environments. The latter study indicated that the changes in expression might be at the base of such phenotypic differences. A fitness advantage gained in mitosis could evolutionarily offset the meiotic costs of the rearrangements.

Inversions involve a change in gene order along the chromosome and are classified into two types: 1) Paracentric inversions which do not encompass the centromere with the inversion breakpoints present on the same arm of the chromosome and 2) pericentric inversions which encompass the centromere with the breakpoints located on different arms of the chromosome. Segregating chromosomal inversions are present in fungi (Perkins 1997; Ohm et al. 2012) and are widespread in higher eukaryotes such as plants (Lowry and Willis 2010), snails (Johannesson and Mikhailova 2003), fruit flies (Feder et al. 2003), rodents (Wise and Pravtcheva 2004), and humans (Stefansson et al. 2005; Zody et al. 2008; Donnelly et al. 2010; Ma and Amos 2012; Salm et al. 2012). They are known to be involved in local adaptation of various species such as *Drosophila melanogaster* (Brehm and Krimbas 1991; Munte et al. 2005; Zivanovic et al. 2014; Kenig et al. 2015) and *Anopheles* (Coluzzi et al. 2002). The process of local adaptation is environment specific and different genes are favored in different environments. Therefore an inversion involving two or more alleles that are adapted to a particular environment will show a selective advantage and hence will spread in the population (Kirkpatrick and Barton 2006; Kirkpatrick 2010). They are also known to have a key role in the coexistence of adaptive phenotypes involving supergenes by maintaining allelic association and reducing recombination and gene flow (Joron et al. 2011).

Inversions have played an important part in evolution of sex chromosomes in mammals. Except for the short pseudoautosomal region, the Y chromosome is completely blocked, and this nonrecombining part of the Y chromosome increased in length due to the number of inversions that occurred during the time of evolution (Lahn and Page 1999). Some studies suggest that this could also be due to local adaptation mechanism, where male and female act as two selective environments. This shows that inversions can also spread in a population by preventing two alleles, which are favored in a particular ecological niche, from recombining (Otto and Lenormand 2002).

Inversions were suggested to be a driving force behind human speciation, although the evidence is controversial (Hey 2003; Navarro and Barton 2003; Zhang et al. 2004). A current genome-wide scan for inversion polymorphisms in human HapMap populations has identified 2,040 candidate inversions and 2 of these (17q21.31 and 8p23.1) have been fully characterized (Stefansson et al. 2005; Zody et al. 2008; Donnelly et al. 2010; Ma and Amos 2012; Salm et al. 2012). The genomes of human and chimpanzees differ by large pericentric inversions and several smaller paracentric rearrangements (Yunis and Prakash 1982; Marques-Bonet et al. 2004). Comparative studies of chimpanzee and human brain cell lines showed that the difference in gene expression between the two genomes is greater in inverted chromosomes compared with the collinear ones, suggesting that the main transcriptional changes occur within the inverted regions (Marques-Bonet et al. 2004). On the other hand, studies on the impact of inversions on the expression profile in *Drosophila* showed that such rearrangements did not have an impact on nearby genes (Meadows et al. 2010).

In *S. cerevisiae*, small inversions within coexpressed gene clusters can induce transcriptional alteration of the neighboring genes. The *DAL* metabolic cluster of *Naumovia castellii* and *S. cerevisiae* differ by two nested inversions involving three genes, *DAL1*, *DAL2*, and *DAL4* (Wong and Wolfe 2005). Our previous study showed that the single inversion of the *DAL2* gene in *S. cerevisiae* reduces the expression of its own gene and that one of the adjacent genes, in addition to compromising the fitness of the invertant colonies (Naseeb and Delneri 2012). At least in some cases, chromosomal inversions are able to disrupt the gene regulatory networks (Jaarola et al. 1998; Goidts et al. 2005), or can promote genes with novel functions (Korneev and O'Shea 2002).

In meiosis, both large and small inversions can be detrimental if the crossing over occurs inside the inversion. Inversions are also responsible for low recombination rates within the breakpoint regions (Dresser et al. 1994; Jaarola et al. 1998; Wallace et al. 2013). Small size inversions can suppress recombination in heterozygote carriers either due to the absence of homosynapsis (Dresser et al. 1994) or the inability to form inversion loops (Jaarola et al. 1998) or by formation of unbalanced gametes carrying deletions and insertions.

Heterozygotes possessing large inversions show very low rate of recombination resulting from double cross overs and gene conversion (Kirkpatrick 2010). Hence, exchange of genetic material within the inverted region is still possible and it can give rise to viable recombinant gametes (Andolfatto et al. 2001). They are therefore thought to be evolutionarily advantageous to the cell.

Chromosomal rearrangements such as translocations are widespread in *S. cerevisiae* sensu stricto species (Fischer et al. 2000) and in natural yeast populations and are believed to contribute to the onset of reproductive isolation (Hou et al. 2014). Small inversions are also widespread in yeast (Seoighe et al. 2000); however, large size chromosomal inversions appear to be less common in these species, compared with higher eukaryotes. So far, no large inversion (over 100 kb) has been reported in yeast, and only one medium size inversion has been described in the natural isolates YJM789, sake yeast, and RM11-1a (∼32.5 kb) (Wei et al. 2007; Akao et al. 2011; Engel and Cherry 2013), and more recently another one in *S*. *arboricola* genome (Liti et al. 2013).

Whether larger inversions encompassing few hundred kilo-bases can be withstood by *S. cerevisiae* genome is not yet clear, as their effect on fitness and gene expression is unknown. Rearrangements usually occur between repetitive regions in the genome and in yeast, *Drosophila* and mosquitoes several chromosomal breakpoints occur near transposons (Mathiopoulos et al. 1998; Evgen'ev et al. 2000; Fischer et al. 2000; Dunham et al. 2002; Garfinkel 2005). Ty elements can cause replication fork stalling resulting in chromosome breakage especially at chromosome fragile sites (Lemoine et al. 2005). They can also undergo homologous recombination with other flanking sequences resulting in the generation of deletions, inversions, and translocations (Downs et al. 1985; Rothstein et al. 1987; Kupiec and Petes 1988). In this work, we created a library of yeast strains carrying all possible peri- and paracentric inversions within the Ty1 elements. We chose to create the inversions within Ty1 elements, because they are the most abundant retrotransposon in the *S. cerevisiae* genome with 31 copies per haploid cell (Kim et al. 1998). Impact of the inversions on reproductive isolation, fitness, and global gene expression has been investigated. We found that some inversions were lethal and, unlike what it was reported for translocations (Avelar et al. 2013), there were no clear mitotic advantages in the 16 invertants tested which could offset the meiotic defects. Interestingly, inversions caused large transcriptional changes without affecting the resulting phenotype, underlining the robustness of the yeast phenotypes.

## Results and Discussion

### Construction of Inverted Strains

We constructed a total of 12 strains (out of 16 attempted rearrangements) carrying pericentric and paracentric inversions of different sizes using the *cre-loxP* recombination system (Delneri et al. 2000). All the inversions were made between yeast transposable elements “Ty1” on different chromosomes in *S. cerevisiae* strain BY4741.

In Stage 1 of construction, a universal Ty insertion cassette having a single *loxP* site with an adjacent antibiotic resistance marker (*loxP-kanMX*), and containing DNA homologous to all Ty1s, was inserted into every Ty1 element without deleting the entire Ty1 sequence. Because the sequences of all 31 Ty1 elements are highly similar, this characteristic was used in our favor and therefore the *loxP* marker cassette was designed to be inserted in the same region in each Ty1 element. We chose to create inversions between Ty elements which were oriented head-to-head or tail-to-tail as only these configurations would give rise to inversions (i.e., Ty1 elements present on chromosomes IV, VII, XIII, XIV, and XVI).

**Fig. 1.**
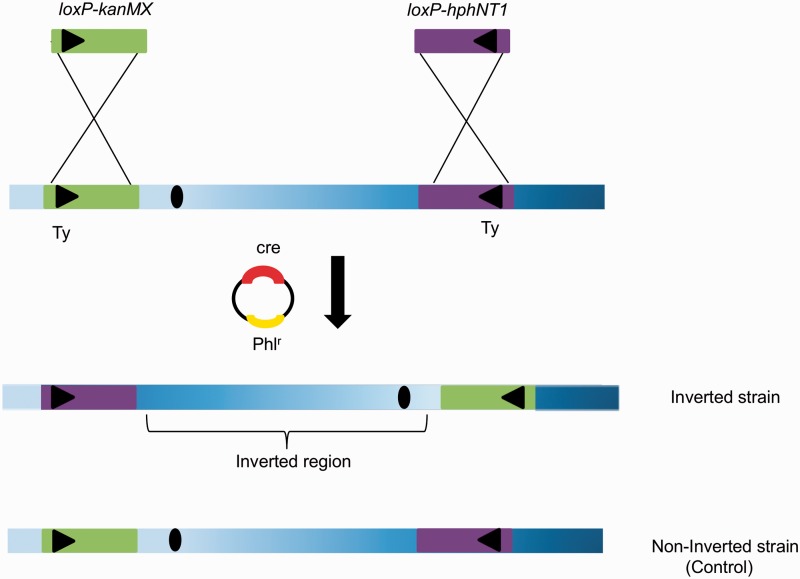
Schematic representation of the construction of inverted and noninverted strains via *cre-loxP* system. The amplified *loxP-kanMX* (green bar) and *loxP-hphNT1* (purple bar) cassettes were inserted in the genome by homologous recombination. Strains possessing the resistant marker cassettes were transformed with the *cre*-containing plasmid pSH cre-ble. Upon induction the cre-recombinase recombines the *lox*P sites (black triangles) and induces the inversion. The blue shaded bar represents the chromosome and the black oval indicates the centromere.

The “Stage 1” strains were used to generate the *loxP-kanMX* + *loxP-hphNT1* strains, which were further used to generate inversions (fig. 1). The Cre plasmid containing the bleomycin resistance marker was introduced in the strains and upon Cre induction, inversions were created. The engineered strains were then restreaked on nonselective medium for several generations in order to lose the Cre plasmid and stabilize the genome structure. Single colonies were checked for the presence or absence of the inversion via polymerase chain reaction (PCR) on purified genomic DNA. For each construct, four different sets of primers were used, specifically binding upstream and downstream of the inversion breakpoints and inside the KanMX or hygromycin marker cassette (supplementary fig. S1*A*–*F*, Supplementary Material online). Furthermore, restriction fragment length polymorphism (RFLP) analysis with different endonucleases was performed to show the different restriction profiles for the noninverted and inverted regions (supplementary fig. S1*G*, Supplementary Material online).

In nature chromosomal inversions will occur among the repetitive elements which are present in head-to-head or tail-to-tail orientation (Downs et al. 1985; Rothstein et al. 1987). Therefore, strains possessing *loxP-kanMX* at Ty1 orientated in opposite direction on the chromosome were chosen to create the inversions. Seven of the constructed strains possessed paracentric inversions and five had pericentric inversions of different sizes on different chromosomes (table 1).

**Table 1.** List of Engineered Strains, Inversion Size, and Genome Location.

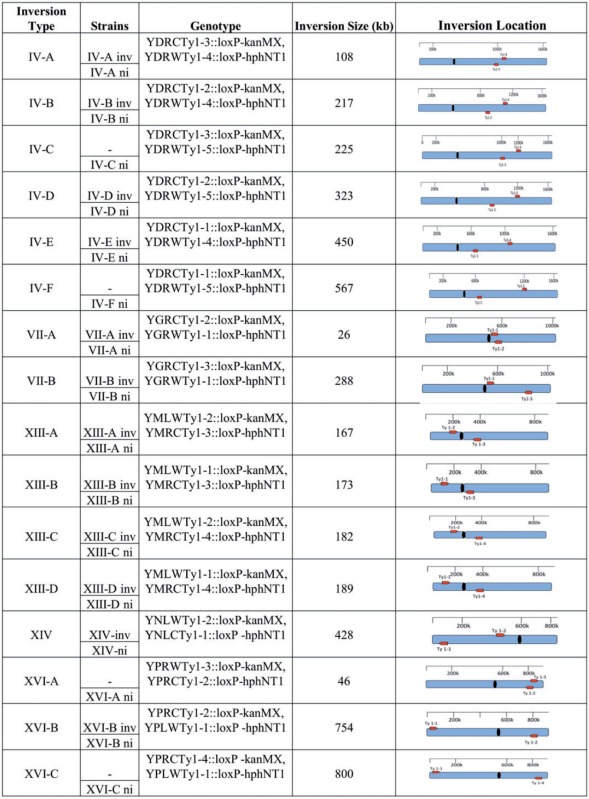

N**ote**. “−” indicates lethal inversion.

### Validation of Lethal Inversions

**Fig. 2.**
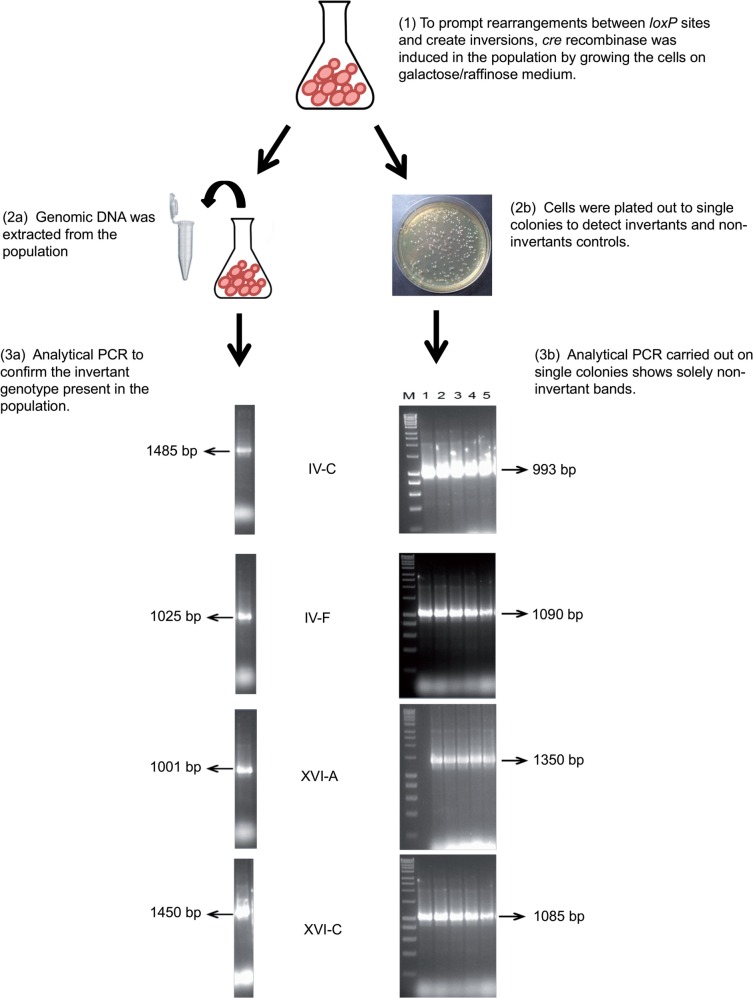
Validation of the presence of lethal inversions in the genome via PCR. The yeast strains IV-C, IV-F, XVI-A, and XVI-C were transformed and induced with *cre* recombinase (1). DNA was extracted from each population of cells after *cre* induction (2a) and a proportion of the same yeast cultures were plated out on YPD medium to allow the growth of single colonies (2b). Analytic PCR was performed to confirm the presence of invertant band in the populations (3a) and in the single growing colonies (3b). The agarose gels show the PCR products corresponding to invertant and noninvertant bands in the strains IV-C, IV-F, XVI-A, and XVI-C. Hyperladder I (10-kb DNA ladder) was used as DNA marker.

In four of our strains (IV-C, IV-F, XVI-A, and XVI-C), inversions could not be obtained (table 1). Usually upon *cre* induction ∼30–50% of the screened colonies would harbor the inversion or translocation depending on the orientation of the *loxP* sites (Delneri et al. 2000; Delneri et al. 2003; Naseeb and Delneri 2012). However, in IV-C, IV-F, XVI-A, and XVI-C all 200 colonies screened contained the noninverted configuration suggesting that either the inversion was not occurring for technical reasons or that it was lethal once established in the cell. To test this hypothesis we extracted the genomic DNA of these strains after *cre* recombinase induction but before plating out the cells. The noninverted and the inverted breakpoint regions were amplified via PCR using the genomic DNA extracted from the population. If the inversion was established in the population, a PCR amplification band corresponding to the inverted region would be detected in our cultures. All our samples showed the inverted band as expected if the inversions were created in the genome (fig. 2).

Because no viable invertant colonies were found after plating out, these data suggest that these specific inversions were lethal, probably interfering with correct mitosis in the haploids.

To test if the lethality of these inversions was a dominant trait, we created heterozygote strains by crossing IV-C, IV-F, XVI-A, and XVI-C (BY4741 background, mat a) with BY4742 (mat alpha). Using the *cre*-recombinase/*loxP* system we induced the inversions in the diploid heterozygotes. The population was tested for the presence of the inversions before and after plating the cells on YPD medium. Upon c*re*-recombinase induction, we were able to amplify the inversion band in all four diploid strains (IV-C, IV-F, XVI-A, and XVI-C); however, no invertant colonies were found after spreading the cells onto the plates (supplementary fig. S2, Supplementary Material online). Overall, these data suggest that these particular inversions, although they can be established in the genome, are dominant lethal. The inversion breakpoints in these strains were located at distance from the telomere and far from the heterochromatic boundary. Therefore, it is unlikely that upon inversion genes located in the euchromatin part of the chromosome (which is transcriptionally more active) were placed in the silent hetrochromatin regions. However, there are few essential and haploinsufficient genes surrounding the breakpoint area and if their expression was altered upon inversion, this may have contributed to the inviable phenotype.

### Spore Viability Is Related to the Numbers of Recombination Hotspots within the Inversion

In meiosis, one or an odd number of cross over events within the inversion has a detrimental effect causing the deletion of part of the genome, and reducing spore viability. Even number of cross overs will result in a swap of DNA within the inversion, with no loss of genetic information. The larger the inversion is, the higher is the probability of recombination occuring.

The number of cross overs that occurs in the yeast genome has been measured experimentally using high-density microarrays and is about 90.5 cross overs per meiosis (Mancera et al. 2008). A later study performed on SK1 × S288C hybrid showed that on average there are ∼2–8 cross over events happening per chromosome (Martini et al. 2011). However, the rate of recombination is also dependent on the size of the chromosomes; the yeast smaller chromosomes undergo more cross overs than the large ones, and this is due to different levels of cross over interference (Kaback et al. 1999).

Earlier studies on chromosomal inversions in Drosophila species reported that big inversions are the ones which are likely to be fixed in a population while the small size ones are rarer (Olvera et al. 1979; Caceres et al. 1997). A more recent study carried out on 12 Drosophila species showed that the genes within the genome have been reorganized due to both micro- and macroinversions (Bhutkar et al. 2008). In *Sz. pombe*, the only two pericentric inversions made by researchers were shown to reduce spore viability to ∼40% in heterozygotic crosses (Avelar et al. 2013).

**Fig. 3.**
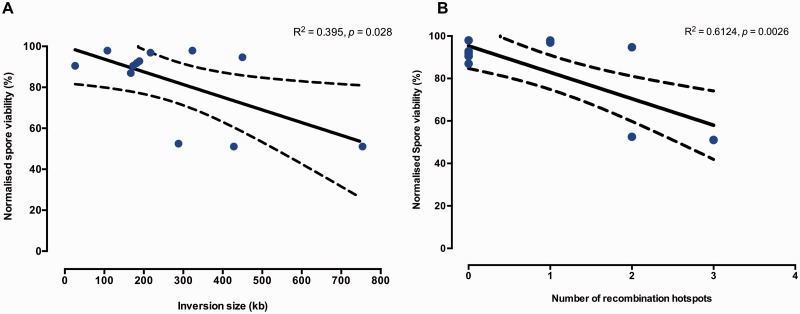
Relationship among normalized spore viability, inversion size, and number of recombination hotspots. Each data point represents the normalized spore viability of each of the invertant strains plotted against the inversion size (*A*) and recombination hotspots (*B*). A small *P* value (<0.05) indicates that the slope of the regression is significantly different from 0, that is, there is a significant correlation between spore viability and size of inversion as well as between spore viability and recombination hotspots.

In order to assess whether the size and position of the inversion on the chromosome had any effect on meiotic fitness, each of the constructed inverted and noninverted strains (control) were crossed with *S. cerevisiae* strain BY4742, and spore viability was assessed. All the inverted strains with exception of three (IV-A.inv, IV-B.inv, and IV-D.inv) when compared with their respective noninverted strains showed a significant drop in spore viability (*P* ≤ 0.05, paired *t*-test; table 2). No relationship was found between the types of inversions and spore viability (slope = −0.389, *P* = 0.38). In fact both pericentric and paracentric inversions seem to have a large range of phenotypic defects from small drop in viability to lethality. In general, the meiotic fitness was related to each specific inversion in case-by-case fashion. Regression analysis on our data only showed a weak negative correlation between the size of inversion and the spore viability (slope = −0.06195, *P* = 0.028; fig. 3*A*).

**Table 2.** List of Engineered Strains Showing the Size of Inversion, Percentage of Spore Viability in Inverted and Noninverted Strains, and Recombination Hotspots.

Strains	Chromosome Bearing Inversion and Size of Chromosome (kb)	Inversion Size (kb)	Spore Viability of Inverted Strain (%)	Spore Viability of Noninverted Strains (%)	Recombination Hotspots
VII-A*	VII (1090)	26	86	95	0
IV-A	IV (1531)	108	95	97	0
XIII-A*^,^^a^	XIII (924)	167	80	92	0
XIII-B*^,^^a^	XIII (924)	173	86	95	0
XIII-C*^,^^a^	XIII (924)	182	90	98	0
XIII-D*^,^^a^	XIII (924)	189	90	97	0
IV-B	IV (1531)	217	94	97	1
VII-B*	VII (1090)	288	52	99	2
IV-D	IV (1531)	323	95	93	1
XIV*	XIV (784)	428	48	94	3
IV-E*	IV (1531)	450	89	94	2
XVI-B*^,^^a^	XVI (948)	754	48	94	3

^a^The strains possessing pericentric inversion (i.e., including the centromere).

*The strains with significant drop in spore viability (*P* ≤ 0.05, paired *t*-test).

Because crossing over is more likely to occur in regions of chromosomes having recombination hotspots (Lichten and Goldman 1995), we corelated our spore viability data with hotspot map. We compared the inversion boundaries of our engineered strains with the genomic co-ordinates of the hotspots using the *S. cerevisiae* map of the cross over hotspot generated by Mancera *et al.* (2008). We observed that all the inverted strains which had one or no recombination hotspots within the inverted region (table 2) had a modest drop in spore viability compared with the noninverted control strains (i.e., ranging from 2% to 12% drop in viable spores), whereas the strains (VII-B, XIV, and XVI-B) with ≥2 recombination hotspots within the inverted region showed 50% drop in spore viability with exception of strain IV-E (fig. 3*B*). Strain IV-E.inv was the only strain that had two recombination hotspots but showed only a small change in spore viability. Regression analysis on this set of data shows a stronger correlation between spore viability and number of hotspots (slope = −12.44, *P* = 0.0026) compared with the size of inversions (fig. 3*A* and *B* and table 2).

### Established Chromosomal Inversions Do Not Have an Impact on the Phenotype in Mitosis

**Fig. 4.**
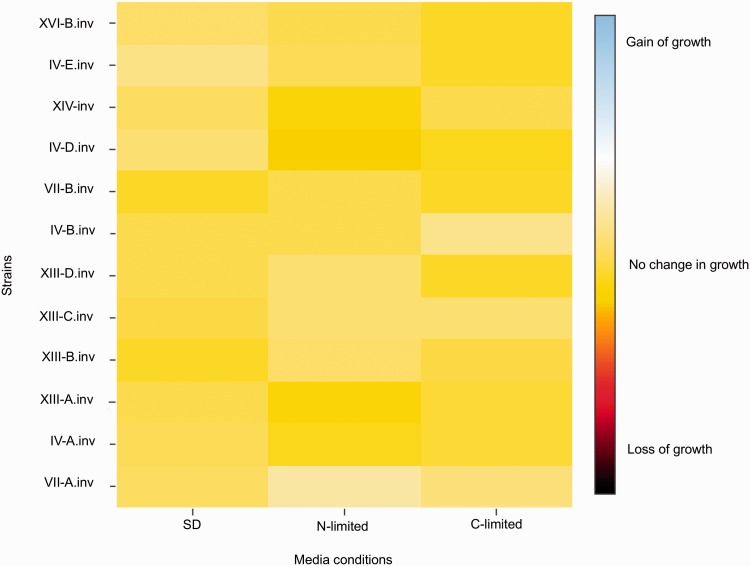
Fitness assays of inverted and noninverted strains in chemically defined media: Significant normalized growth ratio (<10%, *P* > 0.01) of 12 invertant strains compared with the control noninvertants in three nutrient conditions (SD, N-limited, C-limited) is represented using a heatmap with red color indicating loss of growth and blue color for gain of growth.

Chromosomal rearrangements lead to structural and transcriptional changes that can cause growth variability (Colson et al. 2004; Raeside et al. 2014). To study the effect of inversions on growth rate, we checked the fitness of the engineered strains in different nutritional context: synthetic defined (SD) media, chemical defined nitrogen-limited and carbon-limited media (N-limited and C-limited, respectively). We calculated the percentage of fitness variation for each strain carrying inversion compared with its control noninverted isolate in each condition. None of the inversions tested had a detectable effect on the phenotype in *S. cerevisiae* (*P* > 0.05, two-tailed Student’s *t*-test, growth variation <10%; fig. 4).

**Fig. 5.**
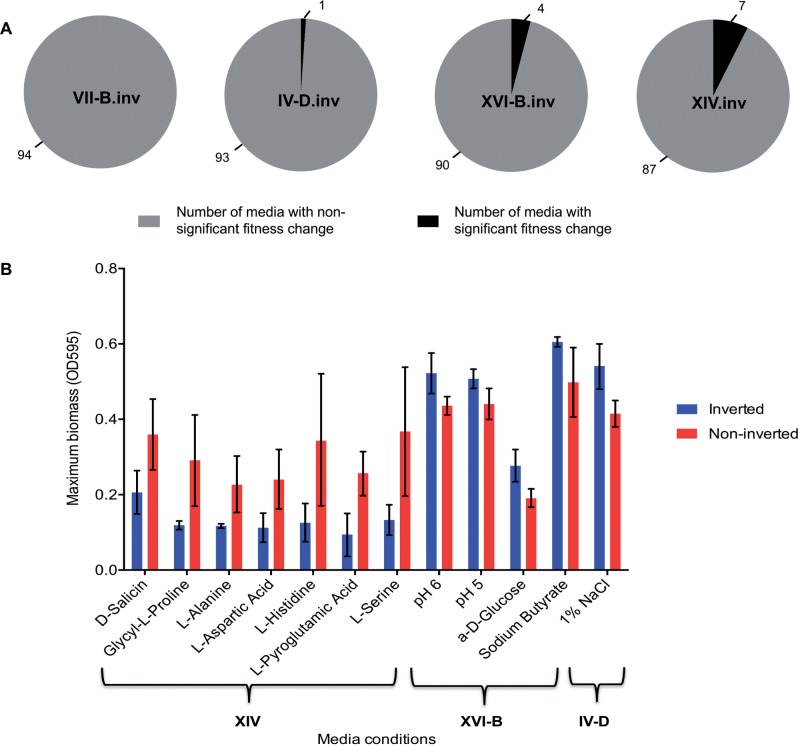
Growth variation of the invertant strains compared with their noninvertant controls in 94 different nutritional conditions: Growth of inverted and noninverted strains XIV, XVI-B, IV-D, and VII-B was measured using Biolog GenIII microtiter plate at 30 °C. (*A*) The total number of media conditions for which significant and nonsignificant growth differences were observed using pie charts with black and gray color indicating the number of significant and nonsignificant media respectively. (B) Significant phenotypic differences (growth ratio > 10%, *P* < 0.01) between inverted and noninverted controls of strains XIV, XVI-B, and IV-D for 12 stress conditions using the bar plot with blue color representing inverted and red color for noninverted strains.

To check the extent of fitness robustness in mitosis, we then performed a larger phenotypic screening encompassing 94 different conditions (Biolog Gen III microtiter plate) on the 3 strains with lowest meiotic fitness (XIV.inv, VII-B.inv, and XVI-B.inv) and 1 strain with no significant drop in spore viability (IV-D.inv; *P* = 0.36). The corresponding noninverted strains were also included in the screening as control. The Biolog plate contains media with different types of sugars as carbon source, different stressful conditions, such as low pH, and different antibiotic stresses (supplementary table S1, Supplementary Material online). There was no significant change in the growth for invertant strain VII-B.inv in any of the 94 media conditions (*P* > 0.01, Mann–Whitney test; fig. 5*A*). The other three strains IV-D.inv, XVI-B.inv, and XIV.inv showed no significant growth difference in 93, 90, and 87 media, respectively (*P* > 0.01, Mann–Whitney test; fig. 5*A*). The strain XIV.inv showed a fitness drop in the remaining seven conditions of which six were environments containing different amino acids such as proline, alanine, aspartic acid, and histidine serine, and pyroglutamic acid (fig. 5*B*). Only in a handful of conditions (i.e., low pH and salt stress) a small increment in final biomass was detected for strains XVI-B.inv and IV-D.inv, respectively (fig. 5*B*). We also carried out competitive fitness assay in SD medium for these four invertant and noninvertant strains relative to the wild type. In all competition studies the phenotypic fitness remained unaltered between the wild type and engineered strains (*P* *>* 0.05, paired *t*-test; supplementary fig. S3, Supplementary Material online).

Once an inversion arises it can spread to become polymorphic and eventually become fixed or be lost. Inversions can evolve either 1) under selection by suppressing recombination during meiosis and locking favorable alleles, as for example in the evolution of resistance to insecticide and desiccation in *Anopheles* sp. (Boussy 1988; Marques-Bonet et al. 2004; Gray et al. 2009; Ayala et al. 2013) or 2) by changing the open reading frames and the transcriptional profile of genes around the breakpoint (Hoffmann and Rieseberg 2008; Kirkpatrick 2010), as for example in bacteria where reversible inversions have been linked to phenotypic alterations, including colony morphology, antibiotic resistance, hemolytic activity, and virulence (Okinaka et al. 2011; Cui et al. 2012). Both these mechanisms can cause fixation of inversions via natural selection. In yeast, the first mechanism is less important because there is little outcrossing (i.e., meiosis happens rarely; Tsai et al. 2008), while the disruption of transcriptional network can be achieved equally effectively by mutations, translocations, and inversions. In both budding and fission yeast, chromosomal translocations can bring an advantage and hence become fixed in a population (Dunham et al. 2002; Colson et al. 2004). One inversion and five translocations in *Sz. pombe* have been reported to be advantageous in mitosis leading the author to conclude that chromosomal rearrangements can be maintained via antagonistic pleiotropy where the disadvantage in meiosis is compensated by an increased mitotic growth (Avelar et al. 2013).

Here, in *S. cerevisiae*, we did not observe significant advantages for the inversions in mitosis in the majority of media tested. In fact one of the strains (XIV.inv) that showed 48% drop in meiotic fitness also had decreased mitotic fitness in 7 of 94 media conditions and possessed a large alteration in transcription profile, whereas strain VII-B.inv showed 52% drop in meiotic fitness but had no change in mitotic fitness in all 94 environments tested. Overall, this supports the idea that large inversions between Ty elements are unlikely to be advantageous and therefore will have little adaptive value. They also have little detrimental effect and may evolve in a neutral fashion.

### Effects of Altered Gene Order on Global Transcription in Inverted and Non-inverted Strains

Transcriptome studies have shown that inversions do not affect the expression of neighborhood genes in *Drosophila* (Meadows et al. 2010); however, in humans the inversion present on chromosome 17q21.31 alters significantly the expression of the genes located both at the breakpoints and inside the inversion (de Jong et al. 2012). Comparative genomic studies between mRNA profile of human and chimpanzee brain cells also uncovered a larger transcriptional difference in rearranged chromosomes when compared with the collinear ones (Marques-Bonet et al. 2004). To determine whether the inversions affect specifically the expression profile of nearby genes, we analyzed the transcriptome on four *S. cerevisiae* inverted strains and compared with their controls. Based on the spore viability data, we selected four inverted strains compared with their respective noninverted controls to determine the effect of the inversions on global gene expression. In particular, we chose all the inverted strains showing a 50% decline in spore viability (VII-B.inv, XIV.inv, and XVI-B.inv) and one strain that had 95% spore viability (IV-D.inv) compared with its respective noninverted partners.

**Fig. 6.**
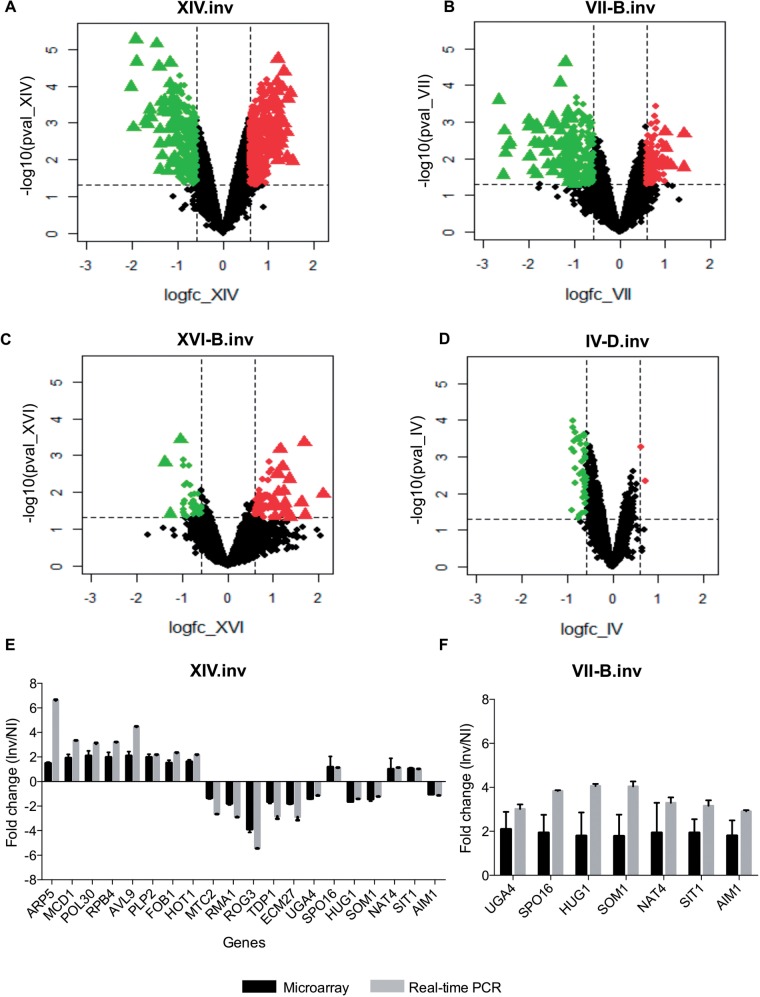
Volcano plot of global expression changes and validation of microarray data. Volcano plots for strains XIV.inv (*A*), VII-B.inv (*B*), XVI-B.inv (*C*), and IV-D.inv (*D*) are reported. The triangles represent the genes with *P* < 0.05 and FC > 2 and the red and green colors indicate the upregulated and downregulated genes, respectively. For strain IV-D.inv, we plotted genes with *P* < 0.05 and FC > 1.5, because no genes with *P* < 0.05 made the cut-off of FC > 2. (*E*, *F*) The validation of microarray data with real-time PCR performed on a subset of genes showing significant change in expression in microarray experiments on strains XIV.inv (*E*) and VII-B.inv (*F*). ΔCt method was applied to calculate the FC in expression and actin was used as a house keeping gene. Error bars were calculated from three technical replicas for each of the three independent biological samples. Black and gray bars represent the FC expression obtained from microarray and qPCR respectively.

Whole transcriptome analysis was performed via hybridization arrays. When comparing the global expression profile between inverted and noninverted strains, genes were considered to be differentially expressed (DE genes) if the *P* value was <0.05 and fold change (FC) > 2 (supplementary data sets S1–S4, Supplementary Material online). The degree to which the expression changes occurred varied according to the type of inversion, with strain XIV.inv showing the highest number of DE genes (fig. 6*A*–*D*). Verification of microarray data was carried out via real-time PCR by validating the expression of 20 and 7 genes in the strains XIV.ni/XIV.inv and VII-B.ni/VII-B.inv, respectively (fig. 6*E* and *F*). The expression levels of tested genes from the microarray and the real-time PCR, across inverted and noninverted strains, were compared using a paired *t*-test to show that there was a high degree of association between the methods (*P* < 0.001). Principal components analysis (PCA) of normalized expression values in log scale for strains VII-B, IV-D, XIV, and XVI-B revealed the relative strength in expression changes due to inversion position (supplementary fig. S4, Supplementary Material online). Plotted on the first two components, strains XIV and VII-B separated clearly on component 1. Strains IV-D and XVI-B were linearly separable in the two components space (PC1 and PC2). A volcano plot of individual gene statistics shows this trend from an alternative perspective (fig. 6).

We found that the global change in the expression varied greatly according to the inversion studied, with inverted strain XIV.inv showing alteration in mRNA profile in ∼13% of its genes (fig. 6*A*), while the rearrangement in strain IV-D.inv has the smallest effect (∼0.4%) on the transcriptome (fig. 6*D*). Although we see important transcriptional changes after inversion, none of the inverted strains showed any growth variation in most of the environments tested (figs. 4 and 5).

**Fig. 7.**
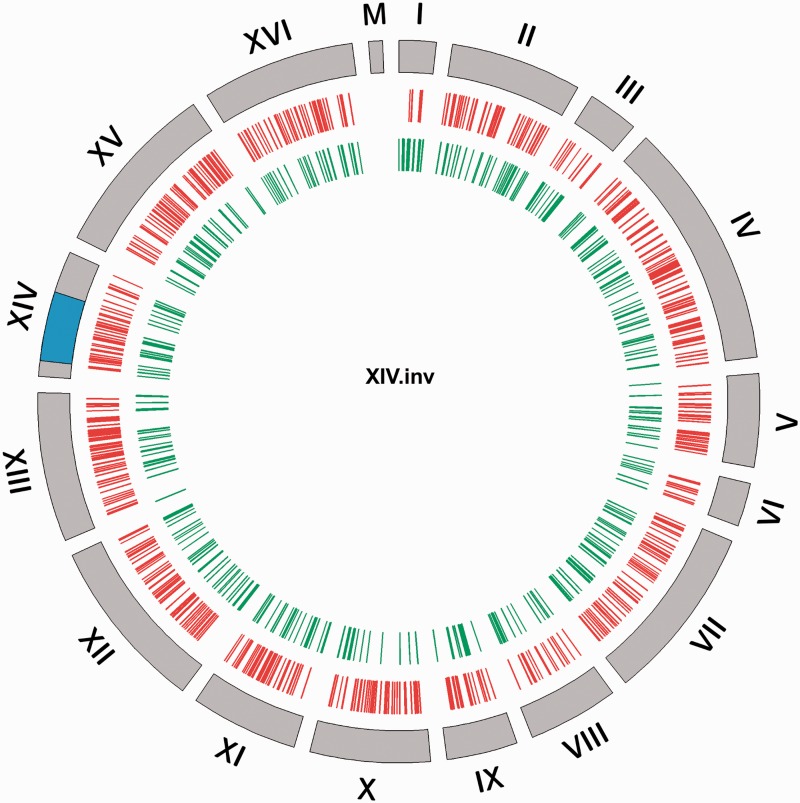
Circus plot of global expression changes in the invertant strain XIV.inv. The DE genes (*q* < 0.05) are reported in red and green color, showing upregulation and downregulation, respectively. The outer gray circle represents the chromosomes and the blue bar indicates the inverted region on the chromosomes.

Our analysis also shows that DE genes were not restricted to the inversion breakpoints or the collinear parts of the chromosomes (fig. 7). Specifically, we analyzed the expression of genes within the inversion breakpoints and in the immediate surrounding regions of the breakpoint (10, 20, and 40 genes on either side of the breakpoints) and compared those with the remaining genome. This was conducted by comparing the significant FC expression between the inverted versus the noninverted strains. A chi-square test and a binomial test were performed on the inverted strains. These showed no significant enrichment of DE genes around the breakpoints and in the inverted region for strains VII-B.inv and strain XVI-B.inv (see supplementary table S2, Supplementary Material online, for *P* values). Strain XIV.inv also showed no significant enrichment of DE genes around the breakpoints; however, an enrichment of DE genes was detected within the inversion (chi-square test: *P* = 0.017 and binomial test: *P* = 0.005; supplementary table S2, Supplementary Material online). Strain IV-D.inv did not have enough transcriptional differences to carry out a robust binomial test. Overall, it appears that the DE genes are scattered around the genome rather than being concentrated in the inversion or nearby the breakpoints (fig. 7).

This expression pattern differs from that seen in brain cell lines of humans and chimpanzees, where genes closer to the breakpoints were shown to have much greater differences in gene expression level compared with those far from the breakpoints, and genes located on rearranged chromosomes had the higher expression differences compared with those present on collinear chromosomes (Marques-Bonet et al. 2004). Our findings are more in line with the study on gene expression in *Drosophila* testis where no preferential change in the expression of adjacent genes in the inverted individuals was detected showing that neighborhood organization is not always a major contributor to gene expression (Meadows et al. 2010).

The inverted strain XIV.inv showed an overall high number of genes changing expression across the genome upon inversion, including few genes important for mitotic growth (i.e., 35 genes, all but 3 upregulated).

The Gene Ontology (GO) analysis on XIV.inv showed a significant enrichment of GO molecular function “structural constituent of nuclear pore” (*P* = 0.00654, hypergeometric distribution) indicating that DNA in the nucleus was more accessible to allow transcriptional changes. Nuclear pore complexes (NPCs) in yeast are composed of ∼30 different nuclear pore proteins known as nucleoporins which exist in 8 or 16 copies per NPC (Rout et al. 2000). The expression analysis of the genes encoding nucleoporins in NPCs “*NUP2*, *NUP60*, *NUP57*, *NUP84*, *NUP116*, *NSP1*, and *GLE1*” showed an upregulation of expression in inverted strain XIV.inv. Mutations in nucleoporins can result in developmental defects and diseases (Saito et al. 2004; Takeda et al. 2006; Zhang et al. 2008) and can also cause changes in chromatin organization and gene regulation (Capelson and Hetzer 2009). Nup2 in yeast interacts with the promoters of active genes during initial steps of transcription (Schmid et al. 2006), and can block the spread of heterochromatin when targeted to the promoter of active genes (Ishii et al. 2002). It has also been shown that a subset of Nups “Nup2 and Nup60” is often located in regions of highly transcribed genes (Casolari et al. 2004). Interestingly, we also observed that the *ARP5* gene located inside the inverted region and involved in chromatin remodeling was significantly upregulated in the inverted strain XIV.inv. We confirmed the differential expression of this gene by real-time PCR which showed a stronger overexpression compared with the control noninverted strain (fig. 6*E*). Arp5 is an important actin-related protein and is a component of INO80 chromatin remodeling complex which plays an important role in DNA replication, transcriptional regulation, and DNA repair (supplementary fig. S5, Supplementary Material online) (Conaway RC and Conaway JW 2009). This complex regulates the transcription of ∼20% of yeast genes. It remodels the nucleosome on promoter to make DNA accessible for transcription (Barbaric et al. 2007; Ford et al. 2007). So, for the XIV.inv strain the global changes can be partially due to the regional effect of the *ARP5* overexpression. We found that *ARP5* was not the only gene in this complex which was DE, but other *INO80* complex genes such as *ARP4* also had altered expression in the inverted strain XIV.inv (supplementary data set S3, Supplementary Material online). Moreover, the transcription of *GCN5* and *HSP26* which are target genes of the INO80 complex was also disrupted in XIV.inv (supplementary data set S3, Supplementary Material online). Changes in the expression of chromatin remodeling genes and nuclear pore genes may be the reason for observing a global extensive transcriptional alteration in strain XIV.inv.

**Fig. 8.**
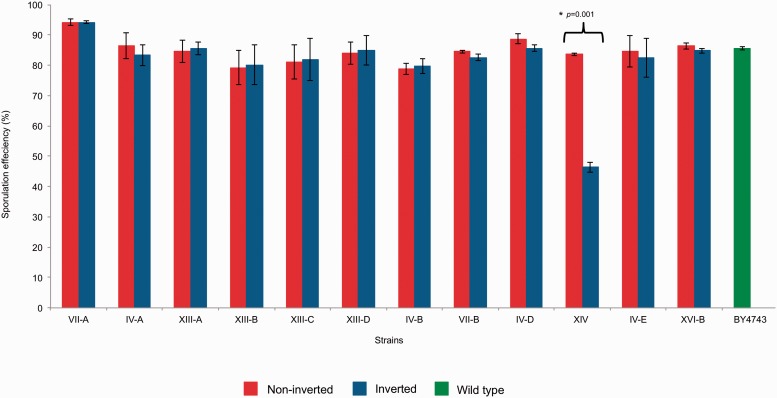
Sporulation efficiency of inverted and noninverted strains. The bar graph shows sporulation efficiency of inverted (blue), noninverted (red), and wild-type BY4743 (green) strains. Each data point represents the average sporulation efficiency (±standard error of the mean) as determined by measuring three biological replicas for each strain.

Our microarray and real-time expression data for strain XIV.inv also showed that *FOB1* and *HOT1* were significantly upregulated (fig. 6*E* and supplementary data set S3, Supplementary Material online). It has been shown that *FOB1* gene product is required for replication fork blocking at RFB sites and recombinational hotspot activity at *HOT1*. Mutants defective in *FOB1* reduce the rates of recombination promoted by *HOT1* (Kobayashi and Horiuchi 1996), and there is a strong correlation between recombination and transcription at the *HOT1* region (Huang and Keil 1995). In fact, overexpression of *FOB1* results in reduced meiotic success (Deutschbauer et al. 2002). Therefore, we anticipate that sporulation efficiency is affected in this inverted strain. We tested XIV.inv for the ability to undergo meiosis, and we found that the sporulation efficiency of the inverted individual was significantly lower when compared with its respective control strain XIV.ni (*P* = 0.001, paired *t*-test), while all the other engineered strains tested showed a similar sporulation efficiency compared with their controls (fig. 8). Therefore, although the mitotic phenotype of this invertant strain is unchanged, there is a disadvantage in meiotic fitness due to a sporulation deficiency.

The GO analysis on the other strains VII-B, XVI-B, and IV-D showed an enrichment of GO molecular functions “glucosyltransferase activity,” “protein kinase activity,” and “enzyme regulator activity,” respectively. Transcriptional regulators are not present in the proximity of the inversion breakpoints for those strains. No essential genes important for mitotic growth changed expression in strain IV-D.inv, while only one gene was downregulated in XVI-B.inv and only two genes were upregulated in strain VII-B.inv.

## Conclusions

Chromosomal rearrangements such as translocations are widespread in *S. cerevisiae* sensu stricto species, and are thought to play an important role in the onset of reproductive isolation (Hou et al. 2015). Genome reorganization not only introduces structural diversity but can also disrupt the regulatory networks controlling the expression of the genes (Raskin et al. 2006; Porcelli et al. 2013). Rearrangements, such as translocations and duplications, can often be associated with rapid adaptation to different environments in yeast (Colson et al. 2004; Chang et al. 2013). Large inversions however seem to be less common in the *Saccharomyces* genus when compared with higher eukaryotes and this raises the question of whether this type of rearrangement is mainly deleterious or does not provide any significant mitotic advantage to the cell.

In this study, we created a library of inverted and noninverted strains having all 16 possible inversions between Ty1 elements (natural substrates for rearrangements) to study how the yeast genome “copes” with such structural changes in terms of fitness, reproductive isolation, and gene expression.

Four of the 16 inversions were apparently lethal, because no viable colonies were detected in a population of cells where the inversion was induced in a diploid background. The other 12 inversions did not provide any mitotic advantage or disadvantage to the invertant yeast cells in rich and nutrient-limited media. In meiosis, 9 of the 12 heterozygote carriers for inversions had significantly lower spore viability when compared with their respective controls, and their fitness defect did not correlate with the type of inversions (table 2). This may be due to the fact that the pericentric inversions constructed did not change greatly the position of the centromere within the chromosome, so in terms of chromosomal structure pericentric and paracentric inversions were not too dissimilar. The two large pericentric inversions still have several essential and nonessential terminal genes that can be affected in mitosis and meiosis, upon inversion. Only a weak correlation was found between spore viability and size of inversion, while a stronger negative correlation was observed between spore viability and the presence of recombination hot spots within the inversion (fig. 3). Regarding the relationship between inversion size and meiotic viability, medium/large inversions are more likely to harbor two crossing over events than smaller inversions and therefore still preserve all the genetic information in meiosis (i.e., no loss of genes and no lethal spores). Double cross overs within medium/large inversions can therefore dampen the effect of the inversion size on the overall spore viability.

Overall, we showed that inversions in yeast can be either deleterious or have a little impact on mitotic fitness, therefore unlikely to offset the meiotic disadvantages. In evolutionary terms, our data suggest that this type of genomic rearrangement is unlikely to be a selectable trait, contrary to what is reported for translocations (Colson et al. 2004; Avelar et al. 2013).

The transcriptional profiles of four invertant strains (three with significant decrease in meiosis and one showing no change) were analyzed and gene expression alterations involving up to 30% of the genome were observed. Interestingly, in all these invertant strains, the transcriptional changes did not translate into a detectable mitotic fitness defect in the 94 nutritional conditions tested (fig. 5). In the strain XIV.inv, with the greatest transcriptional change, the chromatin remodeling gene *ARP5*, placed near the breakpoint, was upregulated 6-fold and this increase could explain the pervasive expression alteration in this strain.

Computer simulation studies on gene regulatory networks predict that the robustness of the transcriptome is important to maintain a normal phenotype in the presence of genetic variation (Wagner 2005). Robustness of the transcriptomic network is in fact considered to be an important buffering system to avoid the deleterious effects of mutations and therefore it is an evolutionarily conserved mechanism (Hartman et al. 2001; Proulx et al. 2007). Here we experimentally showed that, despite the transcriptomic alterations induced by the inversions, fitness remained largely unchanged for all the inverted engineered strains analyzed, suggesting that phenotypic buffering is effective against genetic perturbation. Our results are consistent with studies in *Arabidopsis* where genetic variations visible at a transcriptomic level are neutral in terms of phenotype and do not affect plant performance (Fu et al. 2009; Chan et al. 2012).

Overall, our findings show that alteration of large-scale gene order in *Saccharomyces* species can be deleterious or neutral, and when established have an effect on the transcriptome network although with little consequence on the fitness. The robustness of the yeast phenome is sufficient to maintain homeostasis in the face of transcriptional changes caused by rearrangements.

## Materials and Methods

### Strains, Media, and Culture Conditions

All the Ty inverted and noninverted yeast strains used in this study were constructed in *S. cerevisiae* BY4741 background. A complete list of the strain names and their genotype is given in table 1. The strains were maintained on YPD medium containing 2% (wt./vol.) Bacto-yeast extract, 1% (wt./vol.) Bacto-pepton, and 2% (wt./vol.) glucose. The mineral salt medium (F1 medium: C-limited and N-limited) and SD medium were prepared as described previously (Baganz et al. 1998). The inverted and noninverted strains were sporulated on minimal sporulation medium containing 1% (wt./vol.) potassium acetate and 2% (wt./vol.) Bacto-agar along with the auxotrophic supplements.

### Oligonucleotides

Gene sequences were obtained from Saccharomyces Genome Database (SGD) and PCR primers were designed using the Primer3 program. Primer specificity was checked by the BLAST tool of SGD. All the oligonucleotide sequences are provided in supplementary tables S3–S5, Supplementary Material online.

### Construction of Inverted and Non-inverted Strains

The inverted and noninverted strains were engineered as described previously (Naseeb and Delneri 2012). The resistance gene marker cassettes used in this study were *loxP-kanMX* (Guldener et al. 1996) and *loxP-hphNT1* (Carter and Delneri 2010). The transformants were confirmed for inversion and noninversion by PCR (Delneri et al. 2003). All the primers used for construction of strains are provided in supplementary tables S3 and S4, Supplementary Material online.

### DNA Extraction

Pure cultures of each strain were grown overnight in 5 ml of YPD at 30 °C at 250 rpm on an orbital shaker. Yeast cells were pelleted from a saturated 1.5 ml culture by centrifugation at 10,000 rpm for 5 min. Cell lysis and precipitation of DNA was done using MasterPure yeast DNA purification kit (catalog no. MPY80200) following manufacturer’s instructions. The dry precipitate was resuspended in 35 μl of double-distilled water. DNA was quantified using the nanodrop ultra-low-volume spectrophotometer (Nanodrop Technologies) and the quality was determined by running 0.7% (wt./vol.) agrose gel electrophoresis. The total amount of DNA obtained was ∼2–3 μg/μl. The genomic DNA for PCR was diluted to 5–20 ng/μl.

### Restriction Fragment Length Polymorphism Analysis

PCR products were purified using QIAquick PCR purification kit (catalog no. 28104) following manufacturer’s instructions. A total of 1 μg of PCR product was digested with 1 U of restriction enzyme in 50 μl reaction volume, using the manufacturer’s instructions. The restriction enzymes used were *EcoRI*, *BanII*, *PvuI*, *HindIII*, *AseI*, and *EcoNI* purchased from New England Biolabs. RFLP products were analyzed by gel electrophoresis in 1% (wt./vol.) agarose gels. Hyperladder I (1 kb DNA ladder) was used as a standard DNA marker.

### Spore Viability of Inverted and Non-inverted Strains

Three biological replicas of all inverted and noninverted strains (BY4741 background) were crossed with BY4742 using a microneedle. The hybrids were grown in presporulation medium at 30 °C for 12 h before being plated on minimal sporulation medium. The sporulation plates were incubated at 20 °C for 7–10 days for the formation of tetrads. For each biological replica, 60 tetrads were dissected using Singer MSM-300 micromanipulator. Spore viability was calculated based on the percentage of colonies that had grown for each variant of the strain out of possible 240 dissected spores.

### Measurement of Sporulation Efficiency

For measurement of sporulation efficiency selected inverted and noninverted strains (VII-B, IV-D, XIV, and XVI-B) were crossed with BY4742 to generate diploid hybrids. Three independent biological replicates of each hybrid was sporulated on minimal sporulation medium and assessed for sporulation efficiency as previously described (Tomar et al. 2013). The measurement was done by counting the number of asci produced by ∼200 cells after incubation of 7–10 days at 20 °C.

### Growth Measurement and Competitive Fitness

Growth rate of all the inverted and noninverted strains was determined using FLUOstar optima microplate reader. Cells were grown from a starting OD_595_ of 0.1 to stationary phase in F1 (C- and N-limited) and SD media. The optical density measurements were taken by the microplate reader at 30 °C every 5 min for 48–72 h. Three biological replicates (i.e., different colonies after transformation procedure) were used for each strain. Three technical replicas for each biological replica were included in the screen (nine in total). The readings were blank-corrected using optima data analysis program. Growth rate, maximum biomass, and statistical analysis were calculated using R. The difference in growth was considered as significant if the growth variation between inverted and the noninverted control was >10% and outside the three standard deviation limits with *P* < 0.01 as described previously (Warringer et al. 2003; Hou et al. 2015).

Phenotypic screening was also done using the Biolog Gen III Microplate (catalog no. 1030) in 94 different conditions following the manufacturer’s instructions (Bochner 1989). Maximum growth rate and biomass was calculated using R grofit package (Kahm et al. 2010). Statistical analysis was conducted as described previously (Warringer et al. 2003).

Competitive fitness assays were performed in 96-well plate by adding equal number (5 × 10^5^ cell/ml) of wild-type BY4741 and engineered inverted or noninverted strains in 240 µl of SD medium. The inverted and noninverted strains had KanMX marker which was used for selection between the wild-type and engineered strains. The cultures were grown at 30 °C and maintained in mid-log phase by diluting each culture to 5 × 10^5^ cells/ml in fresh medium every 12 h until generation 50 ± 2 was obtained. The number of generations was calculated as described previously (Parenteau et al. 2008). After 50 generations, ∼200 cells were plated on YPD medium and replica plated on YPD + geneticin to select for the engineered strains. The colonies were counted using Colony Counter SC6+ to obtain the ratio of mutant versus wild-type strains.

### RNA Extraction and Microarray Analysis

Three biological replicas of inverted and noninverted strains were grown in SD medium to mid-log phase (OD_595nm_ = 0.5) and total RNA was extracted in triplicate using Trizol reagent following the manufacturer’s instructions (Invitrogen, catalog no. 155-96-018). RNA was quantified using the nanodrop ultra-low-volume spectrophotometer (Nanodrop Technologies) and the quality of total RNA was determined using Agilent Bioanalyzer 2100 (Agilent Technologies Ltd, UK) whenever required RNA was treated with DNaseI (Fermentas catalog no. EN0521).

Probe preparation and hybridization to Affymetrix GeneChip Yeast Genome 2.0 microarrays were performed according to the manufacturer’s instructions starting with 15 µg of total RNA (Hayes et al. 2007). Technical quality control and outlier analysis were performed with dChip (V2005) (www.dchip.org) using the default settings (Li and Wong 2001). Background correction, quantile normalization, and gene expression analysis were performed using RMA in Bioconductor (Bolstad et al. 2003). The expression patterns within the data set (log scale) were investigated by PCA (DESeq2 [PMID: 25516281] after removal of non-*S. cerevisiae* probesets). The reproducibility of the microarray analysis for each strain was derived from three independent biological replicates in the experiment. Differential expression analysis was performed using Limma using the functions lmFit and eBayes (Smyth 2004). False discovery correction was applied to *P* values to produce a *q* value (Storey and Tibshirani 2003).Three out of four strains had no genes with the cut-off of *q* < 0.05; therefore we also analyzed the data using a more lenient cut-off of *P* < 0.05 and FC > 2. A complete microarray data set was submitted to Minimum Information About a Microarray Experiment (ArrayExpress accession: E-MTAB-2613). The overrepresented GO terms were determined for biological processes for all the significantly expressed genes in inverted strains. The GO analysis was done using the GO Term Finder tool present in SGD (Zivanovic et al. 2014).

### Reverse Transcription and Real-Time Quantitative PCR

Qiagen reverse transcription kit (catalog no. 205311) was used to synthesize cDNA using the random primers following the manufacturer’s instructions. Top ten genes of strain VII-B showing significant change of expression in microarray data set were picked for further validation of expression by real time. A subset of genes showing significant change in expression in strain XIV by microarray was also validated by quantitative PCR (qPCR). Real-time PCR using the Chromo4 gradient thermocycler (biorad) was performed on the cDNA of inverted and noninverted strains using the Quantitect real-time PCR kit from Qiagen (catalog no. 204163). The qPCR conditions were used with an initial denaturation of 3 min at 95 °C followed by 35 cycles consisting of 95 °C for 45 s, 58 °C for 45 s, and 72 °C for 3 min with a final extension of 5 min at 72 °C. Actin (*ACT1*) was used as a housekeeping reference gene and the expression of each gene was estimated using the Ct values. The primers used for real time are shown in supplementary table S5, Supplementary Material online.

## Supplementary Material

Supplementary tables S1–S5, figures S1–S5, data sets S1–S4 are available at *Molecular Biology and Evolution* online (http://www.mbe.oxfordjournals.org).

Supplementary Data

## References

[msw045-B1] Akao T Yashiro I Hosoyama A Kitagaki H Horikawa H Watanabe D Akada R Ando Y Harashima S Inoue T, 2011 Whole-genome sequencing of sake yeast *Saccharomyces cerevisiae* Kyokai no. 7. DNA Res. 18:423–434. 21900213 10.1093/dnares/dsr029 PMC3223075

[msw045-B2] Andolfatto P Depaulis F Navarro A. 2001 Inversion polymorphisms and nucleotide variability in *Drosophila*. Genet Res. 77:1–8. 11279826 10.1017/s0016672301004955

[msw045-B3] Avelar AT Perfeito L Gordo I Ferreira MG. 2013 Genome architecture is a selectable trait that can be maintained by antagonistic pleiotropy. Nat Commun. 4:2235. 23974178 10.1038/ncomms3235

[msw045-B123] Ayala D Guerrero RF Kirkpatrick M. 2013 Reproductive isolation and local adaptation quantified for a chromosome inversion in a malaria mosquito. Evolution. 67:946–958. 23550747 10.1111/j.1558-5646.2012.01836.x

[msw045-B4] Baganz F Hayes A Farquhar R Butler PR Gardner DC Oliver SG. 1998 Quantitative analysis of yeast gene function using competition experiments in continuous culture. Yeast 14:1417–1427. 9848233 10.1002/(SICI)1097-0061(199811)14:15<1417::AID-YEA334>3.0.CO;2-N

[msw045-B5] Barbaric S Luckenbach T Schmid A Blaschke D Horz W Korber P. 2007 Redundancy of chromatin remodeling pathways for the induction of the yeast PHO5 promoter in vivo. J Biol Chem. 282:27610–27621. 17631505 10.1074/jbc.M700623200

[msw045-B6] Bhutkar A Schaeffer SW Russo SM Xu M Smith TF Gelbart WM. 2008 Chromosomal rearrangement inferred from comparisons of 12 *Drosophila* genomes. Genetics 179:1657–1680. 18622036 10.1534/genetics.107.086108 PMC2475759

[msw045-B7] Bochner BR. 1989 Sleuthing out bacterial identities. Nature 339:157–158. 2654644 10.1038/339157a0

[msw045-B8] Bolstad BM Irizarry RA Astrand M Speed TP. 2003 A comparison of normalization methods for high density oligonucleotide array data based on variance and bias. Bioinformatics 19:185–193. 12538238 10.1093/bioinformatics/19.2.185

[msw045-B9] Boutanaev AM Kalmykova AI Shevelyov YY Nurminsky DI. 2002 Large clusters of co-expressed genes in the *Drosophila* genome. Nature 420:666–669. 12478293 10.1038/nature01216

[msw045-B10] Brehm A Krimbas CB. 1991 Inversion polymorphism in *Drosophila obscura*. J Hered. 82:110–117. 2013686 10.1093/oxfordjournals.jhered.a111044

[msw045-B11] Britten RJ. 2002 Divergence between samples of chimpanzee and human DNA sequences is 5%, counting indels. Proc Natl Acad Sci U S A. 99:13633–13635. 12368483 10.1073/pnas.172510699 PMC129726

[msw045-B12] Brown WR Liti G Rosa C James S Roberts I Robert V Jolly N Tang W Baumann P Green C, 2011 A geographically diverse collection of *Schizosaccharomyces pombe* isolates shows limited phenotypic variation but extensive karyotypic diversity. G3 (Bethesda) 1:615–626. 22384373 10.1534/g3.111.001123 PMC3276172

[msw045-B13] Caceres M Barbadilla A Ruiz A. 1997 Inversion length and breakpoint distribution in the *Drosophila buzzath* species complex: is inversion length a selected trait? Evolution 51:1149–1155. 10.1111/j.1558-5646.1997.tb03962.x 28565492

[msw045-B14] Capelson M Hetzer MW. 2009 The role of nuclear pores in gene regulation, development and disease. EMBO Rep. 10:697–705. 19543230 10.1038/embor.2009.147 PMC2727434

[msw045-B15] Carter Z Delneri D. 2010 New generation of loxP-mutated deletion cassettes for the genetic manipulation of yeast natural isolates. Yeast 27:765–775. 20641014 10.1002/yea.1774

[msw045-B16] Casolari JM Brown CR Komili S West J Hieronymus H Silver PA. 2004 Genome-wide localization of the nuclear transport machinery couples transcriptional status and nuclear organization. Cell 117:427–439. 15137937 10.1016/s0092-8674(04)00448-9

[msw045-B17] Chan Z Bigelow PJ Loescher W Grumet R. 2012 Comparison of salt stress resistance genes in transgenic *Arabidopsis thaliana* indicates that extent of transcriptomic change may not predict secondary phenotypic or fitness effects. Plant Biotechnol J. 10:284–300. 22070784 10.1111/j.1467-7652.2011.00661.x

[msw045-B18] Chang SL Lai HY Tung SY Leu JY. 2013 Dynamic large-scale chromosomal rearrangements fuel rapid adaptation in yeast populations. PLoS Genet. 9:e1003232. 23358723 10.1371/journal.pgen.1003232 PMC3554576

[msw045-B19] Cho RJ Campbell MJ Winzeler EA Steinmetz L Conway A Wodicka L Wolfsberg TG Gabrielian AE Landsman D Lockhart DJ, 1998 A genome-wide transcriptional analysis of the mitotic cell cycle. Mol Cell. 2:65–73. 9702192 10.1016/s1097-2765(00)80114-8

[msw045-B20] Cohen BA Mitra RD Hughes JD Church GM. 2000 A computational analysis of whole-genome expression data reveals chromosomal domains of gene expression. Nat Genet. 26:183–186. 11017073 10.1038/79896

[msw045-B21] Colson I Delneri D Oliver SG. 2004 Effects of reciprocal chromosomal translocations on the fitness of *Saccharomyces cerevisiae*. EMBO Rep. 5:392–398. 15105830 10.1038/sj.embor.7400123 PMC1299034

[msw045-B22] Coluzzi M Sabatini A della Torre A Di Deco MA Petrarca V. 2002 A polytene chromosome analysis of the *Anopheles gambiae* species complex. Science 298:1415–1418. 12364623 10.1126/science.1077769

[msw045-B23] Conaway RC Conaway JW. 2009 The INO80 chromatin remodeling complex in transcription, replication and repair. Trends Biochem Sci. 34:71–77. 19062292 10.1016/j.tibs.2008.10.010

[msw045-B24] Csink AK Bounoutas A Griffith ML Sabl JF Sage BT. 2002 Differential gene silencing by trans-heterochromatin in *Drosophila melanogaster*. Genetics 160:257–269. 11805061 10.1093/genetics/160.1.257 PMC1461954

[msw045-B25] Cui L Neoh HM Iwamoto A Hiramatsu K. 2012 Coordinated phenotype switching with large-scale chromosome flip-flop inversion observed in bacteria. Proc Natl Acad Sci U S A. 109:E1647–E1656. 22645353 10.1073/pnas.1204307109 PMC3382547

[msw045-B26] de Jong S Chepelev I Janson E Strengman E van den Berg LH Veldink JH Ophoff RA. 2012 Common inversion polymorphism at 17q21.31 affects expression of multiple genes in tissue-specific manner. BMC Genomics 13:458. 22950410 10.1186/1471-2164-13-458 PMC3582489

[msw045-B27] Delneri D Colson I Grammenoudi S Roberts IN Louis EJ Oliver SG. 2003 Engineering evolution to study speciation in yeasts. Nature 422:68–72. 12621434 10.1038/nature01418

[msw045-B28] Delneri D Tomlin GC Wixon JL Hutter A Sefton M Louis EJ Oliver SG. 2000 Exploring redundancy in the yeast genome: an improved strategy for use of the *cre-loxP* system. Gene 252:127–135. 10903444 10.1016/s0378-1119(00)00217-1

[msw045-B29] Deutschbauer AM Williams RM Chu AM Davis RW. 2002 Parallel phenotypic analysis of sporulation and postgermination growth in *Saccharomyces cerevisiae*. Proc Natl Acad Sci U S A. 99:15530–15535. 12432101 10.1073/pnas.202604399 PMC137751

[msw045-B30] Donnelly MP Paschou P Grigorenko E Gurwitz D Mehdi SQ Kajuna SL Barta C Kungulilo S Karoma NJ Lu RB, 2010 The distribution and most recent common ancestor of the 17q21 inversion in humans. Am J Hum Genet. 86:161–171. 20116045 10.1016/j.ajhg.2010.01.007 PMC2820164

[msw045-B31] Downs KM Brennan G Liebman SW. 1985 Deletions extending from a single Ty1 element in *Saccharomyces cerevisiae*. Mol Cell Biol. 5:3451–3457. 3018520 10.1128/mcb.5.12.3451-3457.1985 PMC369175

[msw045-B32] Dresser ME Ewing DJ Harwell SN Coody D Conrad MN. 1994 Nonhomologous synapsis and reduced crossing over in a heterozygous paracentric inversion in *Saccharomyces cerevisiae*. Genetics 138:633–647. 7851761 10.1093/genetics/138.3.633 PMC1206214

[msw045-B33] Dunham MJ Badrane H Ferea T Adams J Brown PO Rosenzweig F Botstein D. 2002 Characteristic genome rearrangements in experimental evolution of *Saccharomyces cerevisiae*. Proc Natl Acad Sci U S A. 99:16144–16149. 12446845 10.1073/pnas.242624799 PMC138579

[msw045-B34] Elliott CE Callahan DL Schwenk D Nett M Hoffmeister D Howlett BJ. 2013 A gene cluster responsible for biosynthesis of phomenoic acid in the plant pathogenic fungus, *Leptosphaeria maculans*. Fungal Genet Biol. 53:50–58. 23396262 10.1016/j.fgb.2013.01.008

[msw045-B35] Engel SR Cherry JM. 2013 The new modern era of yeast genomics: community sequencing and the resulting annotation of multiple *Saccharomyces cerevisiae* strains at the Saccharomyces Genome Database. Database (Oxford) 2013:bat012. 23487186 10.1093/database/bat012 PMC3595989

[msw045-B36] Evgen'ev MB Zelentsova H Poluectova H Lyozin GT Veleikodvorskaja V Pyatkov KI Zhivotovsky LA Kidwell MG. 2000 Mobile elements and chromosomal evolution in the virilis group of *Drosophila*. Proc Natl Acad Sci U S A. 97:11337–11342. 11016976 10.1073/pnas.210386297 PMC17201

[msw045-B37] Feder JL Roethele JB Filchak K Niedbalski J Romero-Severson J. 2003 Evidence for inversion polymorphism related to sympatric host race formation in the apple maggot fly, *Rhagoletis pomonella*. Genetics 163:939–953. 12663534 10.1093/genetics/163.3.939 PMC1462491

[msw045-B38] Fischer G James SA Roberts IN Oliver SG Louis EJ. 2000 Chromosomal evolution in *Saccharomyces*. Nature 405:451–454. 10839539 10.1038/35013058

[msw045-B39] Fischer G Neuveglise C Durrens P Gaillardin C Dujon B. 2001 Evolution of gene order in the genomes of two related yeast species. Genome Res. 11:2009–2019. 11731490 10.1101/gr.212701

[msw045-B40] Fischer G Rocha EP Brunet F Vergassola M Dujon B. 2006 Highly variable rates of genome rearrangements between hemiascomycetous yeast lineages. PLoS Genet. 2:e32. 16532063 10.1371/journal.pgen.0020032 PMC1391921

[msw045-B41] Ford J Odeyale O Eskandar A Kouba N Shen CH. 2007 A SWI/SNF- and INO80-dependent nucleosome movement at the INO1 promoter. Biochem Biophys Res Commun. 361:974–979. 17681272 10.1016/j.bbrc.2007.07.109 PMC2034749

[msw045-B42] Fu J Keurentjes JJ Bouwmeester H America T Verstappen FW Ward JL Beale MH de Vos RC Dijkstra M Scheltema RA, 2009 System-wide molecular evidence for phenotypic buffering in *Arabidopsis*. Nat Genet. 41:166–167. 19169256 10.1038/ng.308

[msw045-B43] Garfinkel DJ. 2005 Genome evolution mediated by Ty elements in *Saccharomyces*. Cytogenet Genome Res. 110:63–69. 16093659 10.1159/000084939

[msw045-B44] Gierman HJ Indemans MH Koster J Goetze S Seppen J Geerts D van Driel R Versteeg R. 2007 Domain-wide regulation of gene expression in the human genome. Genome Res. 17:1286–1295. 17693573 10.1101/gr.6276007 PMC1950897

[msw045-B45] Goidts V Szamalek JM de Jong PJ Cooper DN Chuzhanova N Hameister H Kehrer-Sawatzki H. 2005 Independent intrachromosomal recombination events underlie the pericentric inversions of chimpanzee and gorilla chromosomes homologous to human chromosome 16. Genome Res. 15:1232–1242. 16140991 10.1101/gr.3732505 PMC1199537

[msw045-B121] Gray EM Rocca KAC Costantini C Besansky NJ 2009 Inversion 2La is associated with enhanced desiccation resistance in *Anopheles gambiae*. Malaria J. 8:215. 10.1186/1475-2875-8-215 PMC2754996 19772577

[msw045-B46] Guldener U Heck S Fielder T Beinhauer J Hegemann JH. 1996 A new efficient gene disruption cassette for repeated use in budding yeast. Nucleic Acids Res. 24:2519–2524. 8692690 10.1093/nar/24.13.2519 PMC145975

[msw045-B47] Hartman JL Garvik B Hartwell L. 2001 Principles for the buffering of genetic variation. Science 291:1001–1004. 11232561 10.1126/science.1056072

[msw045-B48] Hayes A Castrillo JI Oliver SG Brass A Zeef LA. 2007 Transcript analysis: a microarray approach. Methods Microbiol. 36:189–219.

[msw045-B49] Hey J. 2003 Speciation and inversions: chimps and humans. Bioessays 25:825–828. 12938170 10.1002/bies.10336

[msw045-B50] Hoffmann AA Rieseberg LH. 2008 Revisiting the impact of inversions in evolution: from population genetic markers to drivers of adaptive shifts and speciation? Annu Rev Ecol Evol Syst. 39:21–42. 20419035 10.1146/annurev.ecolsys.39.110707.173532 PMC2858385

[msw045-B51] Hou J Friedrich A Gounot JS Schacherer J. 2015 Comprehensive survey of condition-specific reproductive isolation reveals genetic incompatibility in yeast. Nat Commun. 6:7214. 26008139 10.1038/ncomms8214 PMC4445460

[msw045-B52] Hou J Friedrich A Montigny JD Schacherer J. 2014 Chromosomal rearrangements as a major mechanism in the onset of reproductive isolation in *Saccharomyces cerevisiae*. Curr Biol. 24:1153–1159. 24814147 10.1016/j.cub.2014.03.063 PMC4067053

[msw045-B53] Huang GS Keil RL. 1995 Requirements for activity of the yeast mitotic recombination hotspot HOT1: RNA polymerase I and multiple *cis*-acting sequences. Genetics 141:845–855. 8582631 10.1093/genetics/141.3.845 PMC1206849

[msw045-B54] Hurst LD Pal C Lercher MJ. 2004 The evolutionary dynamics of eukaryotic gene order. Nat Rev Genet. 5:299–310. 15131653 10.1038/nrg1319

[msw045-B55] Ishii K Arib G Lin C Van Houwe G Laemmli UK. 2002 Chromatin boundaries in budding yeast: the nuclear pore connection. Cell 109:551–562. 12062099 10.1016/s0092-8674(02)00756-0

[msw045-B56] Jaarola M Martin RH Ashley T. 1998 Direct evidence for suppression of recombination within two pericentric inversions in humans: a new sperm-FISH technique. Am J Hum Genet. 63:218–224. 9634501 10.1086/301900 PMC1377224

[msw045-B57] Johannesson K Mikhailova N. 2003 Habitat-related genetic substructuring in a marine snail (*Littorina fabalis*) involving a tight link between an allozyme and a DNA locus. Biol J Linnean Soc. 81:301–306.

[msw045-B58] Joron M Frezal L Jones RT Chamberlain NL Lee SF Haag CR Whibley A Becuwe M Baxter SW Ferguson L, 2011 Chromosomal rearrangements maintain a polymorphic supergene controlling butterfly mimicry. Nature 477:203–206. 21841803 10.1038/nature10341 PMC3717454

[msw045-B59] Kaback DB Barber D Mahon J Lamb J You J. 1999 Chromosome size-dependent control of meiotic reciprocal recombination in *Saccharomyces cerevisiae*: the role of crossover interference. Genetics 152:1475–1486. 10430577 10.1093/genetics/152.4.1475 PMC1460698

[msw045-B60] Kahm M Hasenbrink G Lichtenberg-Frate H Ludwig J Kschischo M. 2010 grofit: fitting biological growth curves with R. J Stat Softw. 33:1–21.20808728

[msw045-B61] Kellis M Patterson N Endrizzi M Birren B Lander ES. 2003 Sequencing and comparison of yeast species to identify genes and regulatory elements. Nature 423:241–254. 12748633 10.1038/nature01644

[msw045-B62] Kenig B Kurbalija Novicic Z Patenkovic A Stamenkovic-Radak M Andelkovic M. 2015 Adaptive role of inversion polymorphism of *Drosophila subobscura* in lead stressed environment. PLoS One 10:e0131270. 26102201 10.1371/journal.pone.0131270 PMC4478027

[msw045-B63] Kim JM Vanguri S Boeke JD Gabriel A Voytas DF. 1998 Transposable elements and genome organization: a comprehensive survey of retrotransposons revealed by the complete *Saccharomyces cerevisiae* genome sequence. Genome Res. 8:464–478. 9582191 10.1101/gr.8.5.464

[msw045-B64] Kirkpatrick M. 2010 How and why chromosome inversions evolve. PLoS Biol. 8:e1000501. 20927412 10.1371/journal.pbio.1000501 PMC2946949

[msw045-B65] Kirkpatrick M Barton N. 2006 Chromosome inversions, local adaptation and speciation. Genetics 173:419–434. 16204214 10.1534/genetics.105.047985 PMC1461441

[msw045-B66] Kobayashi T Horiuchi T. 1996 A yeast gene product, Fob1 protein, required for both replication fork blocking and recombinational hotspot activities. Genes Cells 1:465–474. 9078378 10.1046/j.1365-2443.1996.d01-256.x

[msw045-B67] Korneev S O'Shea M. 2002 Evolution of nitric oxide synthase regulatory genes by DNA inversion. Mol Biol Evol. 19:1228–1233. 12140234 10.1093/oxfordjournals.molbev.a004183

[msw045-B68] Kupiec M Petes TD. 1988 Allelic and ectopic recombination between Ty elements in yeast. Genetics 119:549–559. 2841187 10.1093/genetics/119.3.549 PMC1203441

[msw045-B69] Lahn BT Page DC. 1999 Four evolutionary strata on the human X chromosome. Science 286:964–967. 10542153 10.1126/science.286.5441.964

[msw045-B70] Lemoine FJ Degtyareva NP Lobachev K Petes TD. 2005 Chromosomal translocations in yeast induced by low levels of DNA polymerase a model for chromosome fragile sites. Cell 120:587–598. 15766523 10.1016/j.cell.2004.12.039

[msw045-B71] Lercher MJ Hurst LD. 2006 Co-expressed yeast genes cluster over a long range but are not regularly spaced. J Mol Biol. 359:825–831. 16631793 10.1016/j.jmb.2006.03.051

[msw045-B72] Li C Wong WH. 2001 Model-based analysis of oligonucleotide arrays: expression index computation and outlier detection. Proc Natl Acad Sci U S A. 98:31–36. 11134512 10.1073/pnas.011404098 PMC14539

[msw045-B73] Lichten M Goldman AS. 1995 Meiotic recombination hotspots. Annu Rev Genet. 29:423–444. 8825482 10.1146/annurev.ge.29.120195.002231

[msw045-B74] Liti G Nguyen Ba AN Blythe M Muller CA Bergstrom A Cubillos FA Dafhnis-Calas F Khoshraftar S Malla S Mehta N, 2013 High quality de novo sequencing and assembly of the *Saccharomyces arboricolus* genome. BMC Genomics 14:69. 23368932 10.1186/1471-2164-14-69 PMC3599269

[msw045-B75] Lowry DB Willis JH. 2010 A widespread chromosomal inversion polymorphism contributes to a major life-history transition, local adaptation, and reproductive isolation. PLoS Biol. 8:e1000500. 20927411 10.1371/journal.pbio.1000500 PMC2946948

[msw045-B76] Ma J Amos CI. 2012 Investigation of inversion polymorphisms in the human genome using principal components analysis. PLoS One 7:e40224. 22808122 10.1371/journal.pone.0040224 PMC3392271

[msw045-B77] Mancera E Bourgon R Brozzi A Huber W Steinmetz LM. 2008 High-resolution mapping of meiotic crossovers and non-crossovers in yeast. Nature 454:479–485. 18615017 10.1038/nature07135 PMC2780006

[msw045-B78] Marques-Bonet T Caceres M Bertranpetit J Preuss TM Thomas JW Navarro A. 2004 Chromosomal rearrangements and the genomic distribution of gene-expression divergence in humans and chimpanzees. Trends Genet. 20:524–529. 15475109 10.1016/j.tig.2004.08.009

[msw045-B79] Martini E Borde V Legendre M Audic S Regnault B Soubigou G Dujon B Llorente B. 2011 Genome-wide analysis of heteroduplex DNA in mismatch repair-deficient yeast cells reveals novel properties of meiotic recombination pathways. PLoS Genet. 7:e1002305. 21980306 10.1371/journal.pgen.1002305 PMC3183076

[msw045-B80] Mathiopoulos KD della Torre A Predazzi V Petrarca V Coluzzi M. 1998 Cloning of inversion breakpoints in the *Anopheles gambiae* complex traces a transposable element at the inversion junction. Proc Natl Acad Sci U S A. 95:12444–12449. 9770505 10.1073/pnas.95.21.12444 PMC22850

[msw045-B81] Meadows LA Chan YS Roote J Russell S. 2010 Neighbourhood continuity is not required for correct testis gene expression in *Drosophila*. PLoS Biol. 8:e1000552. 21151342 10.1371/journal.pbio.1000552 PMC2994658

[msw045-B82] Munte A Rozas J Aguade M Segarra C. 2005 Chromosomal inversion polymorphism leads to extensive genetic structure: a multilocus survey in *Drosophila subobscura*. Genetics 169:1573–1581. 15687280 10.1534/genetics.104.032748 PMC1449531

[msw045-B83] Naseeb S Delneri D. 2012 Impact of chromosomal inversions on the yeast DAL cluster. PLoS One 7:e42022. 22916115 10.1371/journal.pone.0042022 PMC3419248

[msw045-B84] Navarro A Barton NH. 2003 Chromosomal speciation and molecular divergence–accelerated evolution in rearranged chromosomes. Science 300:321–324. 12690198 10.1126/science.1080600

[msw045-B85] Ohm RA Feau N Henrissat B Schoch CL Horwitz BA Barry KW Condon BJ Copeland AC Dhillon B Glaser F, 2012 Diverse lifestyles and strategies of plant pathogenesis encoded in the genomes of eighteen *Dothideomycetes* fungi. PLoS Pathog. 8:e1003037. 23236275 10.1371/journal.ppat.1003037 PMC3516569

[msw045-B86] Okinaka RT Price EP Wolken SR Gruendike JM Chung WK Pearson T Xie G Munk C Hill KK Challacombe J, 2011 An attenuated strain of *Bacillus anthracis* (CDC 684) has a large chromosomal inversion and altered growth kinetics. BMC Genomics 12:477. 21962024 10.1186/1471-2164-12-477 PMC3210476

[msw045-B87] Olvera O Powell JR De La Rosa ME Salceda VM Gaso MI. 1979 Population genetics of Mexican *Drosophila* VI. Cytogenetic aspects of the inversion polymorphism in *Drosophila pseudoobscura*. Evolution 33:381–395. 10.1111/j.1558-5646.1979.tb04691.x 28568185

[msw045-B88] Otto SP Lenormand T. 2002 Resolving the paradox of sex and recombination. Nat Rev Genet. 3:252–261. 11967550 10.1038/nrg761

[msw045-B89] Parenteau J Durand M Veronneau S Lacombe AA Morin G Guerin V Cecez B Gervais-Bird J Koh CS Brunelle D, 2008 Deletion of many yeast introns reveals a minority of genes that require splicing for function. Mol Biol Cell. 19:1932–1941. 18287520 10.1091/mbc.E07-12-1254 PMC2366882

[msw045-B90] Perkins DD. 1997 Chromosome rearrangements in *Neurospora* and other filamentous fungi. Adv Genet. 36:239–398. 9348657 10.1016/s0065-2660(08)60311-9

[msw045-B91] Porcelli I Reuter M Pearson BM Wilhelm T van Vliet AH. 2013 Parallel evolution of genome structure and transcriptional landscape in the Epsilonproteobacteria. BMC Genomics 14:616. 24028687 10.1186/1471-2164-14-616 PMC3847290

[msw045-B92] Proulx SR Nuzhdin S Promislow DE. 2007 Direct selection on genetic robustness revealed in the yeast transcriptome. PLoS One 2:e911. 17878946 10.1371/journal.pone.0000911 PMC1975671

[msw045-B93] Raeside C Gaffe J Deatherage DE Tenaillon O Briska AM Ptashkin RN Cruveiller S Medigue C Lenski RE Barrick JE, 2014 Large chromosomal rearrangements during a long-term evolution experiment with *Escherichia coli*. MBio 5:e01377–e01314. 25205090 10.1128/mBio.01377-14 PMC4173774

[msw045-B94] Raskin DM Seshadri R Pukatzki SU Mekalanos JJ. 2006 Bacterial genomics and pathogen evolution. Cell 124:703–714. 16497582 10.1016/j.cell.2006.02.002

[msw045-B95] Rothstein R Helms C Rosenberg N. 1987 Concerted deletions and inversions are caused by mitotic recombination between delta sequences in *Saccharomyces cerevisiae*. Mol Cell Biol. 7:1198–1207. 3550432 10.1128/mcb.7.3.1198-1207.1987 PMC365193

[msw045-B96] Rout MP Aitchison JD Suprapto A Hjertaas K Zhao Y Chait BT. 2000 The yeast nuclear pore complex: composition, architecture, and transport mechanism. J Cell Biol. 148:635–651. 10684247 10.1083/jcb.148.4.635 PMC2169373

[msw045-B97] Saito S Miyaji-Yamaguchi M Nagata K. 2004 Aberrant intracellular localization of SET-CAN fusion protein, associated with a leukemia, disorganizes nuclear export. Int J Cancer. 111:501–507. 15239126 10.1002/ijc.20296

[msw045-B98] Salm MP Horswell SD Hutchison CE Speedy HE Yang X Liang L Schadt EE Cookson WO Wierzbicki AS Naoumova RP, 2012 The origin, global distribution, and functional impact of the human 8p23 inversion polymorphism. Genome Res. 22:1144–1153. 22399572 10.1101/gr.126037.111 PMC3371712

[msw045-B99] Schmid M Arib G Laemmli C Nishikawa J Durussel T Laemmli UK. 2006 Nup-PI: the nucleopore-promoter interaction of genes in yeast. Mol Cell. 21:379–391. 16455493 10.1016/j.molcel.2005.12.012

[msw045-B100] Seoighe C Federspiel N Jones T Hansen N Bivolarovic V Surzycki R Tamse R Komp C Huizar L Davis RW, 2000 Prevalence of small inversions in yeast gene order evolution. Proc Natl Acad Sci U S A. 97:14433–14437. 11087826 10.1073/pnas.240462997 PMC18936

[msw045-B101] Singer GA Lloyd AT Huminiecki LB Wolfe KH. 2005 Clusters of co-expressed genes in mammalian genomes are conserved by natural selection. Mol Biol Evol. 22:767–775. 15574806 10.1093/molbev/msi062

[msw045-B102] Smyth GK. 2004 Linear models and empirical bayes methods for assessing differential expression in microarray experiments. Stat Appl Genet Mol Biol. 3: Article3. 10.2202/1544-6115.1027 16646809

[msw045-B103] Stefansson H Helgason A Thorleifsson G Steinthorsdottir V Masson G Barnard J Baker A Jonasdottir A Ingason A Gudnadottir VG, 2005 A common inversion under selection in Europeans. Nat Genet. 37:129–137. 15654335 10.1038/ng1508

[msw045-B104] Storey JD Tibshirani R. 2003 Statistical significance for genomewide studies. Proc Natl Acad Sci U S A. 100:9440–9445. 12883005 10.1073/pnas.1530509100 PMC170937

[msw045-B105] Takeda A Goolsby C Yaseen NR. 2006 NUP98-HOXA9 induces long-term proliferation and blocks differentiation of primary human CD34+ hematopoietic cells. Cancer Res. 66:6628–6637. 16818636 10.1158/0008-5472.CAN-06-0458

[msw045-B106] Teichmann SA Veitia RA. 2004 Genes encoding subunits of stable complexes are clustered on the yeast chromosomes: an interpretation from a dosage balance perspective. Genetics 167:2121–2125. 15342545 10.1534/genetics.103.024505 PMC1471008

[msw045-B107] Tomar P Bhatia A Ramdas S Diao L Bhanot G Sinha H. 2013 Sporulation genes associated with sporulation efficiency in natural isolates of yeast. PLoS One 8:e69765. 23874994 10.1371/journal.pone.0069765 PMC3714247

[msw045-B108] Tsai IJ Bensasson D Burt A Koufopanou V. 2008 Population genomics of the wild yeast *Saccharomyces paradoxus*: quantifying the life cycle. Proc Natl Acad Sci U S A. 105:4957–4962. 18344325 10.1073/pnas.0707314105 PMC2290798

[msw045-B109] Wagner A. 2005 Robustness, evolvability, and neutrality. FEBS Lett. 579:1772–1778. 15763550 10.1016/j.febslet.2005.01.063

[msw045-B110] Wallace AG Detweiler D Schaeffer SW. 2013 Molecular population genetics of inversion breakpoint regions in *Drosophila pseudoobscura*. G3 (Bethesda) 3:1151–1163. 23665879 10.1534/g3.113.006122 PMC3704243

[msw045-B111] Warringer J Ericson E Fernandez L Nerman O Blomberg A. 2003 High-resolution yeast phenomics resolves different physiological features in the saline response. Proc Natl Acad Sci U S A. 100:15724–15729. 14676322 10.1073/pnas.2435976100 PMC307635

[msw045-B112] Wei W McCusker JH Hyman RW Jones T Ning Y Cao Z Gu Z Bruno D Miranda M Nguyen M, 2007 Genome sequencing and comparative analysis of *Saccharomyces cerevisiae* strain YJM789. Proc Natl Acad Sci U S A. 104:12825–12830. 17652520 10.1073/pnas.0701291104 PMC1933262

[msw045-B113] Wise TL Pravtcheva DD. 2004 Oligosyndactylism mice have an inversion of chromosome 8. Genetics 168:2099–2112. 15611179 10.1534/genetics.104.031914 PMC1448711

[msw045-B122] Wolfe KH Shields DC. 1997 Molecular evidence for an ancient duplication of the entire yeast genome. Nature. 387:708–713. 9192896 10.1038/42711

[msw045-B114] Wong S Wolfe KH. 2005 Birth of a metabolic gene cluster in yeast by adaptive gene relocation. Nat Genet. 37:777–782. 15951822 10.1038/ng1584

[msw045-B115] Xu Z Wei W Gagneur J Perocchi F Clauder-Munster S Camblong J Guffanti E Stutz F Huber W Steinmetz LM. 2009 Bidirectional promoters generate pervasive transcription in yeast. Nature 457:1033–1037. 19169243 10.1038/nature07728 PMC2766638

[msw045-B116] Yunis JJ Prakash O. 1982 The origin of man: a chromosomal pictorial legacy. Science 215:1525–1530. 7063861 10.1126/science.7063861

[msw045-B117] Zhang J Wang X Podlaha O. 2004 Testing the chromosomal speciation hypothesis for humans and chimpanzees. Genome Res. 14:845–851. 15123584 10.1101/gr.1891104 PMC479111

[msw045-B118] Zhang X Chen S Yoo S Chakrabarti S Zhang T Ke T Oberti C Yong SL Fang F Li L, 2008 Mutation in nuclear pore component NUP155 leads to atrial fibrillation and early sudden cardiac death. Cell 135:1017–1027. 19070573 10.1016/j.cell.2008.10.022

[msw045-B119] Zivanovic G Arenas C Mestres F. 2014 Inversion polymorphism in two Serbian natural populations of *Drosophila subobscura*: analysis of long-term changes. Genetika 50:638–644. 25715453 10.7868/s0016675814060150

[msw045-B120] Zody MC Jiang Z Fung HC Antonacci F Hillier LW Cardone MF Graves TA Kidd JM Cheng Z Abouelleil A, 2008 Evolutionary toggling of the MAPT 17q21.31 inversion region. Nat Genet. 40:1076–1083. 19165922 10.1038/ng.193 PMC2684794

