# Supplementary material for: Widespread Impact of Chromosomal Inversions on Gene Expression Uncovers Robustness via Phenotypic Buffering
Source: Mol Biol Evol. 2016 Feb 28;33(7):1679–96. doi: 10.1093/molbev/msw045 (PMC4915352; doi:10.1093/molbev/msw045)
Supplement: Supplementary Data [file supp_33_7_1679__index.html]

Widespread Impact of Chromosomal Inversions on Gene Expression Uncovers Robustness via Phenotypic Buffering — Widespread Impact of Chromosomal Inversions on Gene Expression Uncovers Robustness via Phenotypic Buffering — Supplementary Data 

# Widespread Impact of Chromosomal Inversions on Gene Expression Uncovers Robustness via Phenotypic Buffering

## Supplementary Data

files

- Supplementary Data - zip file
