# Supplementary material for: Widespread Impact of Chromosomal Inversions on Gene Expression Uncovers Robustness via Phenotypic Buffering
Source: Mol Biol Evol. 2016 Feb 28;33(7):1679–96. doi: 10.1093/molbev/msw045 (PMC4915352; doi:10.1093/molbev/msw045)
Supplement: Supplementary Data [file supp_msw045_suppl_data.zip › MBE resub Supplementary data file SN_DD.pdf]

Supplementary Information

Widespread impact of chromosomal inversions on gene expression uncovers robustness via phenotypic buffering.

Samina Naseeb, Zorana Carter, David Minnis, Ian Donaldson, Leo Zeef, and Daniela Delneri

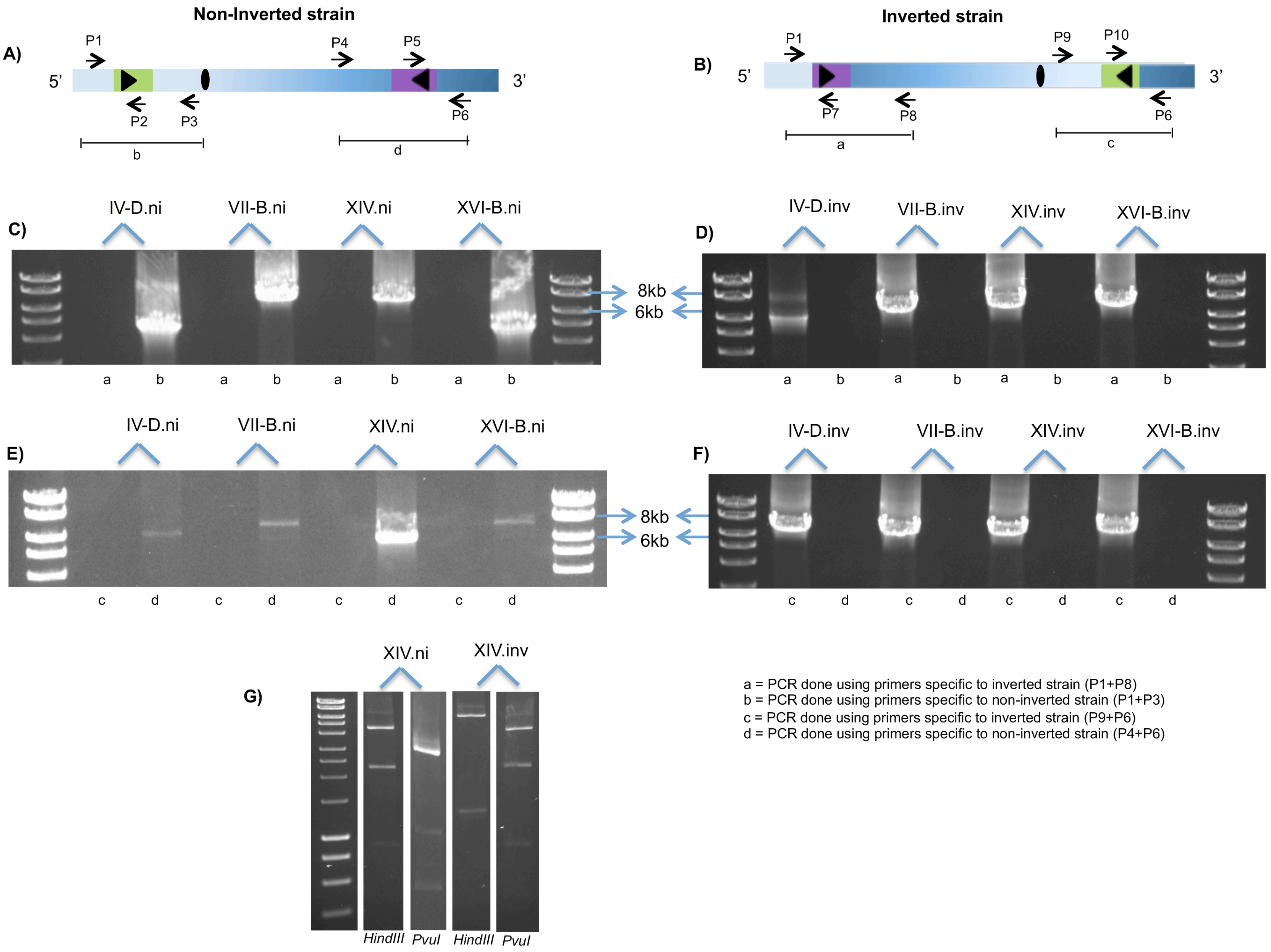

a = PCR done using primers specific to inverted strain (P1+P8)
b = PCR done using primers specific to non-inverted strain (P1+P3)
c = PCR done using primers specific to inverted strain (P9+P6)
d = PCR done using primers specific to non-inverted strain (P4+P6)

Figure S1: Validation of the non-inverted and inverted strains via PCR and RFLP. Panels A and B: Schematic representation of the non-inverted and inverted strains along with the specific primer binding sites. The green box is the KanMX marker with one lox P (black arrow) and the purple box is the Hygromycin marker with one loxP (black arrow). Upon inversion the markers swap position in the genome (panel B). Panels C and D: Diagnostic Long Range Analytical PCR was performed using the primers binding upstream and downstream the inversion breakpoint at the 5' end of the chromosome to confirm the presence of non-invertant and invertant strains respectively. Panels E and F) Diagnostic Long Range Analytical PCR was performed using the primers binding upstream and downstream the inversion breakpoint at the 3' end of the chromosome to confirm the presence of non-invertant and invertant strains respectively. Each strain was checked by using both sets of primers specific to the inverted (a and c) and non-inverted (b and d) regions. Panel G) Different restriction profiles of the non-inverted and inverted strain XIV were obtained by digesting the two long PCR products with *HindIII* (breakpoint near the 5' end of the chromosome) and *PvuI* (breakpoint near the 3'end of chromosome) enzymes. Hyperladder I (10-kb DNA ladder) was used as DNA marker.

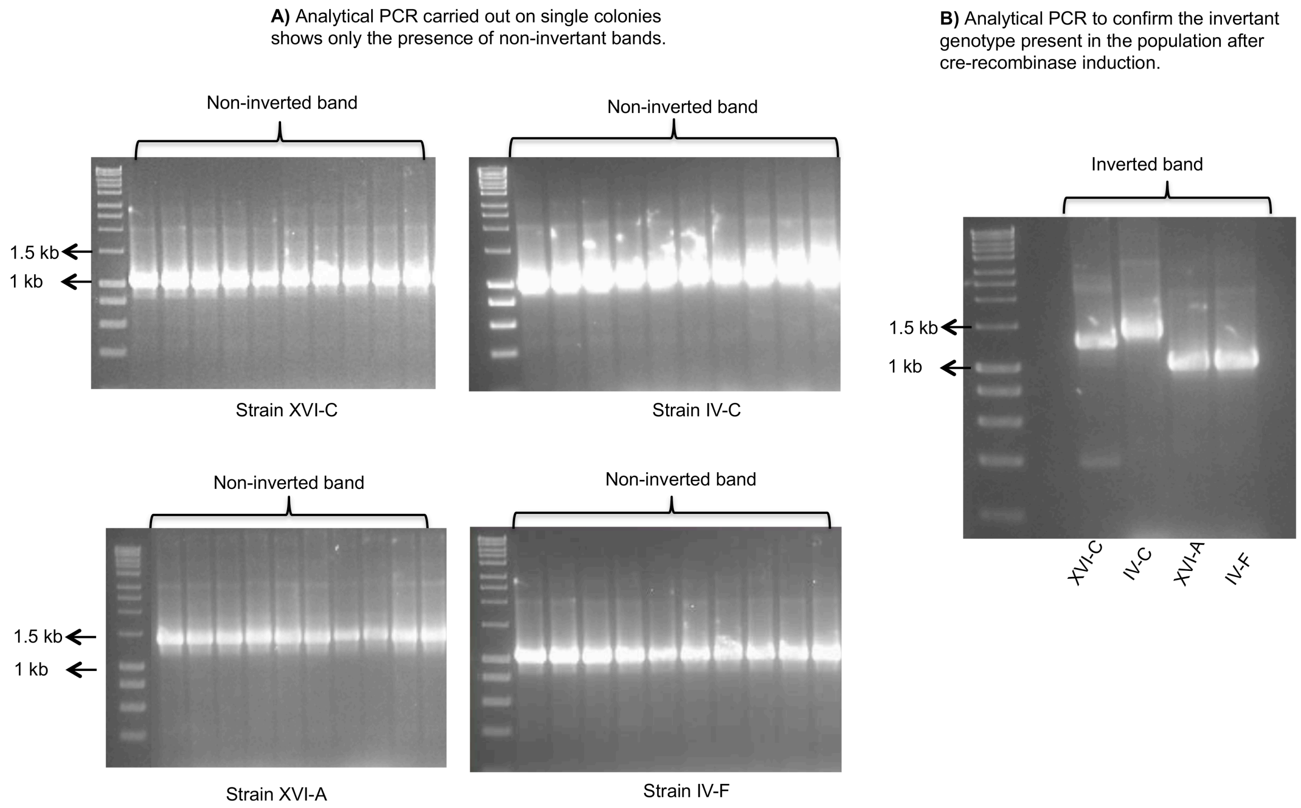

Figure S2: Validation of the presence of lethal inversions in heterozygote strains via PCR. The diploid yeast strains XVI-C, IV-C, XVI-A and IV-F were transformed and induced with *cre* recombinase. Panel A: Analytical PCR was performed on the single growing colonies, which showed only the presence of non-invertant bands. Panel B: Analytical PCR was performed on the genomic DNA extracted from the yeast population after the induction of Cre recombinase to induce the inversion. The presence of invertant the band in the population was confirmed. The agarose gels show the PCR products corresponding to invertant and non-invertant bands in the strains XVI-C, IV-C, XVI-A and IV-F. Hyperladder I (10-kb DNA ladder) was used as DNA marker.

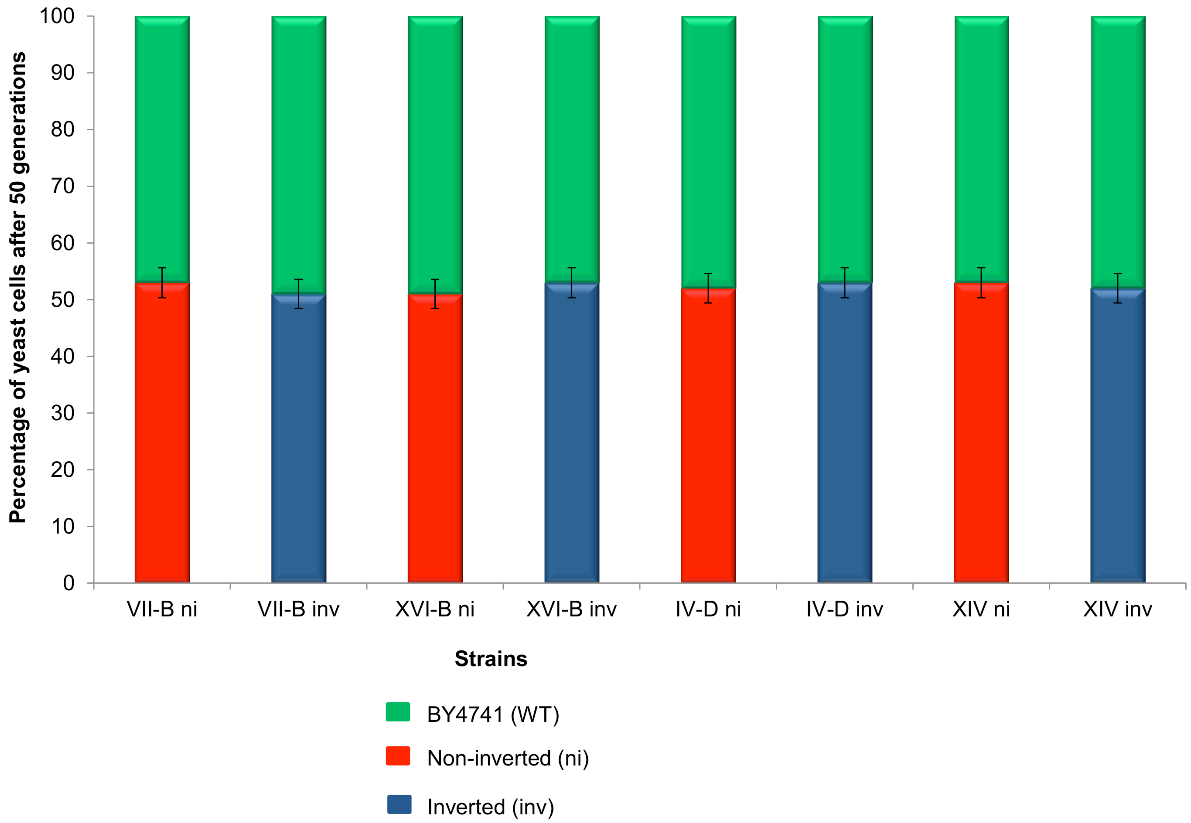

Figure S3: Competitive growth assay of wild type (BY4741), inverted and non-inverted strains in SD medium. Mixed cultures were started by adding equal quantities of cells from each strain (WT + inverted and WT + non-inverted) in SD medium. After 50 generations, ~200 cells were plated on YPD medium and replica plated on YPD + geneticin to select for the inverted and non-inverted strains. The error bars represent the average of three technical replicas of three independent biological replicas. Error bars are at 95% confidence intervals. Green, red and blue bars represent the wild type, non-inverted and inverted strains respectively.

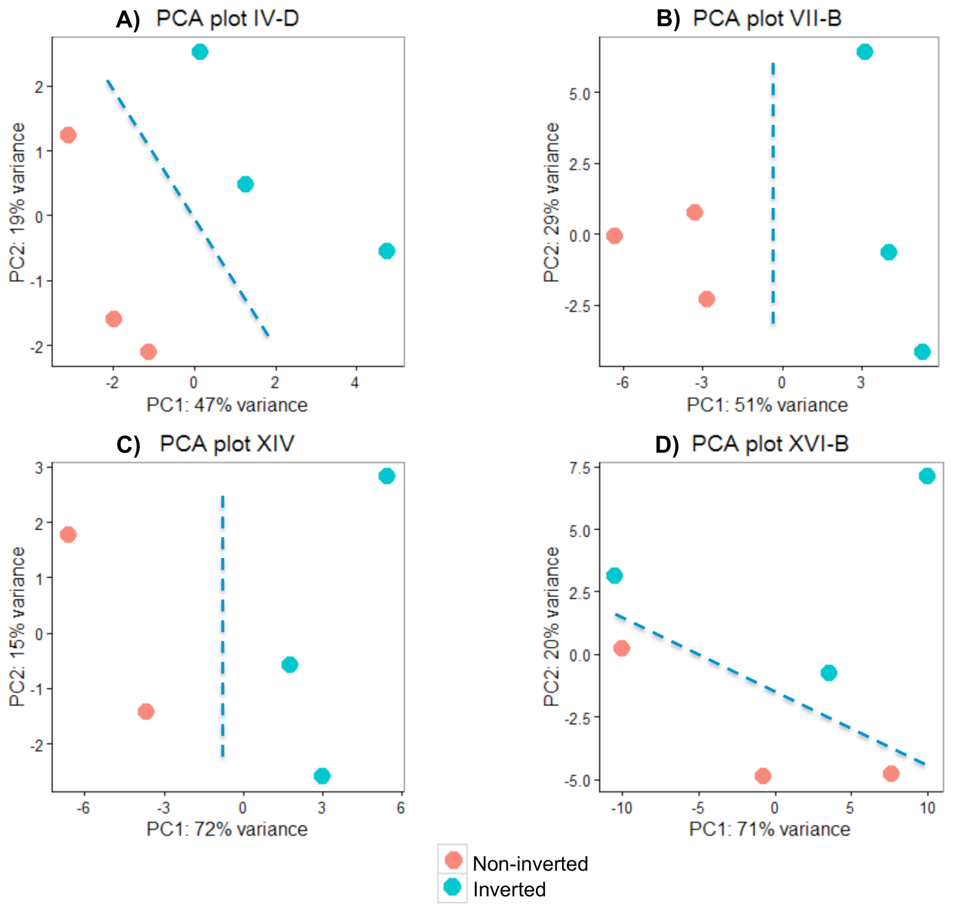

Figure S4: Principal component analysis (PCA) of microarray data set. The Figure represents the overall variation for the three inverted replicas (blue) and three non-inverted replicas (red), where each spot represents an individual array. Each Panel represents PCA analysis for strains IV-D (panel A), VII-B (panel B), XIV (panel C) and XVI-B (panel D) respectively. Strains VII-B (panel B) and XIV (panel C) separated clearly on component 1. Strains IV-D and XVI-B were linearly separable in the two components space (PC1 and PC2). For strain XIV only two biological replicates for the non-inverted strain are reported due to technical issues.

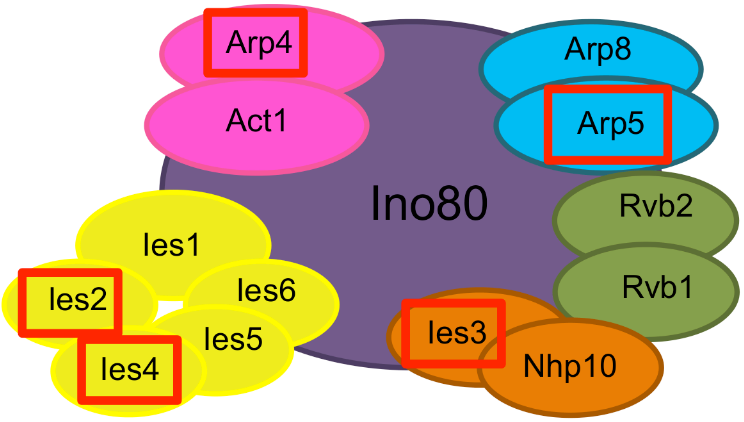

Figure S5: Structure of the INO80 remodelling complex. Diagrammatic view of INO80 complex consisting of inositiol requiring protein Ino80, actin Act1, actin related protein Arp4 and Arp5 and other proteins Ies1, Ies2, Ies3, Ies4, Ies5, Ies6, Rvb1 and Rvb2. Red box indicates the genes with significant differential expression in inverted strain XIV.

Supplementary Tables

Table S1. The 96 well lay out for Biolog Gen III microtitre plate.

	1	2	3	4	5	6	7	8	9	10	11	12
A	Negative Control	Dextrin	D-Maltose	D-Trehalose	D-Cellobiose	Gentiobiose	Sucrose	D-Turanose	Stachyose	Positive Control	pH 6	pH 5
B	D-Raffinose	α-D-Lactose	D-Melibiose	β-Methyl-DGlucoside	D-Salicin	N-Acetyl-DGlucosamine	N-Acetyl-β-DMannosamine	N-Acetyl-DGalactosamine	N-Acetyl Neuraminic Acid	1% NaCl	4% NaCl	8% NaCl
C	α-D-Glucose	D-Mannose	D-Fructose	D-Galactose	3-Methyl Glucose	D-Fucose	L-Fucose	L-Rhamnose	Inosine	1% Sodium	Fusidic Acid	D-Serine
D	D-Sorbitol	D-Mannitol	D-Arabitol	myo-Inositol	Glycerol	D-Glucose-6-PO4	D-Fructose-6-PO4	D-Aspartic Acid	D-Serine	Troleandomycin	Rifamycin SV	Minocycline
E	Gelatin	Glycyl-L-Proline	L-Alanine	L-Arginine	L-Aspartic Acid	L-Glutamic Acid	L-Histidine	L-Pyroglutamic Acid	L-Serine	Lincomycin	Guanidine HCl	Niaproof 4
F	Pectin	D-Galacturonic Acid	L-Galactonic Acid Lactone	D-Gluconic Acid	D-Glucuronic Acid	Glucuronamide	Mucic Acid	Quinic Acid	D-Saccharic Acid	Vancomycin	Tetrazolium Violet	Tetrazolium Blue
G	p-Hydroxy-Phenylacetic Acid	Methyl Pyruvate	D-Lactic Acid Methyl Ester	L-Lactic Acid	Citric Acid	α-Keto-Glutaric Acid	D-Malic Acid	L-Malic Acid	Bromo-Succinic Acid	Nalidixic Acid	Lithium Chloride	Potassium Tellurite
H	Tween 40	γ-Amino-Butryric Acid	α-Hydroxy-Butyric Acid	β-Hydroxy-D,Lbutyric Acid	α-Keto-Butyric Acid	Acetoacetic Acid	Propionic Acid	Acetic Acid	Formic Acid	Aztreonam	Sodium Butyrate	Sodium Bromate

Table S2: Summary of the binomial and Chi-square tests

	Region pvalue<0.05 and FC≥1.5				All other genes not in region being tested				Test Results	
	Number List	Number DE	Fraction	Number Not DE	Number List	Number DE	Fraction	Number Not DE	Pvalue binomial	Pvalue Chi square (Pearson's)
VII-B_10 genes either side bp	40	1	0.025	39	5676	302	0.053	5374	0.252	0.428
VII-B_20 genes either side bp	80	3	0.038	77	5636	300	0.053	5336	0.184	0.533
VII-B_40 genes either side bp	160	10	0.063	150	5556	293	0.053	5263	0.112	0.587
VII-B_within inversion	127	10	0.079	117	5589	293	0.052	5296	0.060	0.191
XIV_10 genes either side bp	40	5	0.125	35	5676	666	0.117	5010	0.185	0.881
XIV_20 genes either side bp	80	9	0.113	71	5636	662	0.117	4974	0.139	0.891
XIV_40 genes either side bp	160	19	0.119	141	5556	652	0.117	4904	0.097	0.957
XIV_within inversion	213	36	0.169	177	5503	635	0.115	4868	0.005*	0.017*
XVI-B_10 genes either side bp	40	1	0.025	39	5676	90	0.016	5586	0.340	0.645
XVI-B_20 genes either side bp	80	2	0.025	78	5636	89	0.016	5547	0.228	0.513
XVI-B_40 genes either side bp	160	2	0.013	158	5556	89	0.016	5467	0.254	0.726
XVI- B_within inversion	363	7	0.019	356	5353	84	0.016	5269	0.131	0.597

* represents the *p* values <0.05; bp refers to "breakpoints".

Table S3. The set of cassette amplifying primers for engineering inverted and non-inverted strains

Primer Name	Sequence 5'-3'	Tm(°C)
TY1_F	TCATGGTAGCGCCTGTGCTTCGGTTACTTCTAAGGAAGTCCACAGCTGAAGCTTCGTACG	70.9
TY1_R	GCGTTAATTGGTATATTTCTTTTTTGAGCATCAACTACGTTTATGTAAGGGTTCTCGAGAGCTCG	66.7

Table S4. List of checking primers used for confirming inverted and non-inverted strains

Strains	Primer name	Sequence 5'-3'
IV-A	P1-F	CTGAGTAGGCGAGGCAGG
	P3-R	TCACAATGGAATCCCAACAATTACATCAA
	P4-F	GCGTTGCGGTTTTCAGTATT
	P6-R	TTGCCTTGTAGGTCCGAAAC
	P8-R	GCCTTGAAGTACCCACCAGA
	P9-F	TCATTTCGCACATAGCTTGC
IV-B	P1-F	AAATTGTCGGGATATTAGTG
	P3-R	CAAGAAATGCCAAGGTGGAT
	P4-F	GCGTTGCGGTTTTCAGTATT
	P6-R	TTGCCTTGTAGGTCCGAAAC
	P8-R	GCCTTGAAGTACCCACCAGA
	P9-F	TATCCCACAAGGTGGTCCAT
IV-C (lethal)	P1-F	CTGAGTAGGCGAGGCAGG
	P3-R	TCACAATGGAATCCCAACAATTACATCAA
	P4-F	TCACTTTCTCCGGACGATTC
	P6-R	TTATAGCGTTTGCCGGAAAC
IV-D	P1-F	AAATTGTCGGGATATTAGTG
	P3-R	CAAGAAATGCCAAGGTGGAT
	P4-F	TCACTTTCTCCGGACGATTC
	P6-R	TTATAGCGTTTGCCGGAAAC
	P8-R	GGCCTGGGTGTCAATACACT
	P9-F	TCTGTGTTGACCGTGGGTTA
IV-E	P1-F	GGGGGCAAGCTTACTTCAA
	P3-R	GAGTTTTCGTCCGCATCAAT
	P4-F	GCGTTGCGGTTTTCAGTATT
	P6-R	TTGCCTTGTAGGTCCGAAAC

	P8-R	GCCTTGAAGTACCCACCAGA
	P9-F	TGCGTTTACCAACAGTGGAA
IV-F (lethal)	P1-F	GGGGGCAAGCTTACTTCAA
	P3-R	GAGTTTTCGTCCGCATCAAT
	P4-F	TCACTTTCTCCGGACGATTC
	P6-R	TTATAGCGTTTGCCGGAAAC
VII-A	P1-F	ACACTAGATACTCTACTCGACG
	P3-R	GATTTCCGCCTGTCCAGTAA
	P4-F	TGTTGGCACCAACCAATC
	P6-R	GGTGCTGTTGTCCCAAAGTT
	P8-R	GCTTTAGGCAACAACCGAAA
	P9-F	GCGATCCACGAACTTCATTT
VII-B	P1-F	ACACTAGATACTCTACTCGACG
	P3-R	GATTTCCGCCTGTCCAGTAA
	P4-F	AAGGACGCACAAAAATACGG
	P6-R	AAGCTGAAACGCAAGGATTG
	P8-R	AACGCAAAGTCGAAGGTGAT
	P9-F	GCGATCCACGAACTTCATTT
XIII-A	P1-F	CTTAGCCACTAAGGAATCACC
	P3-R	CCCGTGCTGACCGTATCTAT
	P4-F	ATCTCACCATCCCTGGACAC
	P6-R	GGGAAAAGCCGAGCTTTAGT
	P8-R	AGCCGAAAAGGGCTAGGTAA
	P9-F	GGAGACAGAGCGGCTTAGAA
XIII-B	P1-F	TATGCGCTGCATCTTGTGG
	P3-R	AAGGCCGATACATTGTCGAG
	P4-F	ATCTCACCATCCCTGGACAC
	P6-R	GGGAAAAGCCGAGCTTTAGT
	P8-R	AGCCGAAAAGGGCTAGGTAA
	P9-F	TTCCTAGGGTCCCCAAGTTT

XIII-C	P1-F	CTTAGCCACTAAGGAATCACC
	P3-R	CCCGTGCTGACCGTATCTAT
	P4-F	AAGCCCTTGCGAACAATAGA
	P6-R	TCAGCGGTGTTTTTGCAC
	P8-R	GTCTTGCAGGGGTCAAGCTA
	P9-F	GGAGACAGAGCGGCTTAGAA
XIII-D	P1-F	TATGCGCTGCATCTTGTGG
	P3-R	AAGGCCGATACATTGTCGAG
	P4-F	AAGCCCTTGCGAACAATAGA
	P6-R	TCAGCGGTGTTTTTGCAC
	P8-R	GTCTTGCAGGGGTCAAGCTA
	P9-F	TTCCTAGGGTCCCCAAGTTT
XIV	P1-F	ATATGTGTGGGAAATGGGTG
	P3-R	AGCCTAGCAAAAGCTGATCG
	P4-F	GACGCCTGGAGTCAAAGAAG
	P6-R	AAACCAGGGCTGATGATTTG
	P8-R	ACAAGTTACATCGCGGGTTC
	P9-F	GGAGTGACCCCAGCAAATAA
XVI-A (lethal)	P1-F	CGGAAGTGCCGCGATACTAG
	P3-R	ATTGCCGTAATTTTCCATGC
	P4-F	TTCAATGGCAATGGCTTACA
	P6-R	CACGTGACAACTGCGAGAGT
XVI-B	P1-F	TCGACCATCACGTAGTAGTC
	P3-R	AACTCGGCAGAGCTTGTCAT
	P4-F	CGGAAGTGCCGCGATACTAG
	P6-R	ATTGCCGTAATTTTCCATGC
	P8-R	GCGAAACGCAAGACTCTCTC
	P9-F	ACGCAAAGTCGAAGGTGATT
XVI-C (lethal)	P1-F	TCGACCATCACGTAGTAGTC
	P3-R	AACTCGGCAGAGCTTGTCAT

	P4-F	GAGCACTTGCAGCAATACGA
	P6-R	TGCCATATTTTTGCACCGTA
Universal	P2 (KAN)-R	AACGTGAGTCTTTTCCTTACC
	P5 (HPH)-F	AGCATCAGCTCATC GAGAG
	P7 (HPH)-R	ACCTGCCTGAAACCGAACT
	P10 (KAN)-F	TCGTCACTCATGGTGATTTC

Table S5. List of primers used for Real Time PCR.

Primer Name	Sequence 5'-3'	Tm (°C)
UGA4-F	CATGCCTGCTGTCTGGACTA	60
UGA4-R	GCAAAGCCGTACTTGGAGTC	59
NAT4-F	CGTGCCCGTGATATACCTTT	59
NAT4-R	TAGAACCCAAGCGCCTCATA	60
SPO16-F	AAAGCTAACCAGCGTTTGACA	59
SPO16-R	AAAGCAACCACCAAGCTTTC	59
SIT1-F	ATGGGGACCATCATTGAGTC	59
SIT1-R	TTACGTTCCCGCTAATCCAG	60
AIM1-F	CAATGACTCCGGAGGAGAAG	59
AIM-R	AATTGTAGGCCATGCCATCT	59
SOM1-F	AGCAACGCTTGACTCACAAA	59
SOM1-R	TGTGTCCGTGACCTCGTAGT	59
HUG1-F	ACCAAGGCCTTAACCCAAAG	60
HUG1-R	GTTCGACGGCAATGATGTT	59
ARP5-RT-F	ACCGAAAGACTGGCAACTGT	59
ARP5-RT-R	GCACCATCAACTACGGGAAT	59
MCD1-F	CCACAGCCCACCAATTTTAC	55
MCD1-R	CCCTTCTCGCCCATTATGTA	55
POL30-F	CGGTGATATCGGATCAGGTT	55
POL30-R	AAGGGAGGAGCCCTTAATGA	55
RPB4-F	AACCGTCGGGGCAGTTATAC	58
RPB4-R	AAAGAACCTAGTTGCGCCAC	56
AVL9-F	GACCAGGAAAGCATCGAGTC	56
AVL9-R	TTCTGTATCGCGTTCGATGT	55
PLP2-F	TTACCGGGGTGAGGTAATCA	55

PLP2-R	ACAGCGCCAACTTTTACCAT	56
FOB1-F	TGGCTTGGGTTTGAAGAGTT	55
FOB1-R	TTAGGCACTTACCCCAATCG	55
HOT1-F	TCACTGCCCACTCACATTTC	56
HOT1-R	GACTGGTACCTGTGGGTGCT	59
MTC2-F	GTGGTACTTCCCCATGCACT	57
MTC2-R	GGCGGCACGACTTTACTTAG	57
RMA1-F	AAGTTCGCCTGCCATACAGT	57
RMA1-R	GAGCTGCCTTCGCATTATTT	55
ROG3-F	TTAAAGGGGTCCTCCTCACC	56
ROG3-R	GCTGCGTTCAATAACGGATT	55
TDP1-F	AATATGGCTCCGGTCAACAG	55
TDP1-R	TTAAAAACGGCACCTTTTGC	54
ECM27-F	ACGTTTATTGTGCCGAGCTT	56
ECM27-R	GGACGCCGTTTTTGAGACTA	56
